# Current global vitamin and cofactor prescribing practices for primary mitochondrial diseases: Results of a European reference network survey

**DOI:** 10.1002/jimd.12805

**Published:** 2024-11-11

**Authors:** Julia Neugebauer, Karit Reinson, Marcello Bellusci, Julien H. Park, Omar Hikmat, Enrico Bertini, Manuel Schiff, MetabERN PM‐MD Consortium authors, Anna Ardissone, Niklas Darin, Alejandra Darling, Daria Diodato, Luisa Diogo, Erle Kristensen, Beata Kieć‐Wilk, Maria Carmo Macário, Diego Martinelli, Martina Messina, Mar O'Callaghan, Juan Darío Ortigoza‐Escobar, Margarida Paiva Coelho, Leticia Pías, Jolanta Sykut‐Cegielska, Arnaud Vanlander, Shamima Rahman

**Affiliations:** ^1^ Department of Paediatric Gastroenterology Nephrology and Metabolic Medicine, Charité – Universitaetsmedizin Berlin Berlin Germany; ^2^ Center for Chronically Sick Children Charité – Universitaetsmedizin Berlin Berlin Germany; ^3^ Department of Clinical Genetics, Genetics and Personalized Medicine Clinic Tartu University Hospital Tartu Estonia; ^4^ Department of Genetics and Personalized Medicine, Institute of Clinical Medicine University of Tartu Tartu Estonia; ^5^ Reference Center for Inherited Metabolic Disorders MetabERN Mitochondrial Disorders Research Group (imas12) ‘12 de Octubre’ University Hospital Madrid Spain; ^6^ Department of General Paediatrics University Hospital Muenster Muenster Germany; ^7^ Department of Paediatrics and Adolescent Medicine Haukeland University Hospital Norway; ^8^ Department of Clinical Medicine (K1) University of Bergen Norway; ^9^ Research Unit of Neuromuscular and Neurodegenerative Disease, Translational Pediatrics and Clinical Genetics Bambino Gesu' Children's Hospital, IRCCS Rome Italy; ^10^ Université Paris Cité Institut Imagine, Genetics of Mitochondrial Disorders, INSERM UMR Paris France; ^11^ Reference Centre for Mitochondrial Disorders and Reference Centre for Metabolic Disease, AP‐HP Necker‐Enfants Malades Hospital Paris France; ^12^ Mitochondrial Research Group, Genetics and Genomic Medicine Department UCL Great Ormond Street Institute of Child Health London UK; ^13^ Metabolic Unit Great Ormond Street Hospital for Children NHS Foundation Trust London UK

**Keywords:** cofactors, cross sectional study, inherited metabolic disease, primary mitochondrial disease, survey, treatment, vitamins

## Abstract

Primary mitochondrial diseases (PMD) account for a group of approximately 400 different genetic disorders with diverse clinical presentations and pathomechanisms. Although each individual disorder is rare, collectively they represent one of the largest groups in the field of inherited metabolic disorders. The complexity of PMD results in a continued lack of therapeutic options, necessitating a predominantly symptomatic treatment approach for affected patients. While a subset of diseases responds exceptionally well to treatment with specific vitamins or cofactors, for most PMD systematic reviews were not able to show significant benefit. This is in discrepancy to their continued frequent use among specialists. To gain further insight into the current clinical practice of vitamin and cofactor supplementation among clinicians treating children and adults affected by PMD, we conducted a worldwide cross‐sectional questionnaire study exploring the choice of substances and the specific diseases where they are applied. To our knowledge, this is the first global study exploring this topic and featuring a high response rate from paediatricians. The vast majority (95%, 106/112) of responding specialists recommended the use of vitamins and cofactors, either in an agnostic approach irrespective of the specific PMD or directed to the treatment of specific diseases or phenotypes. Our study highlights significant regional and specialty‐specific differences in supplementation practices. We provide some preliminary insights into specialist‐based opinions regarding the use of vitamins and cofactors in PMD and highlight the need for more rigorous clinical and preclinical investigations and/or clear consensus statements.

## Graphical abstract

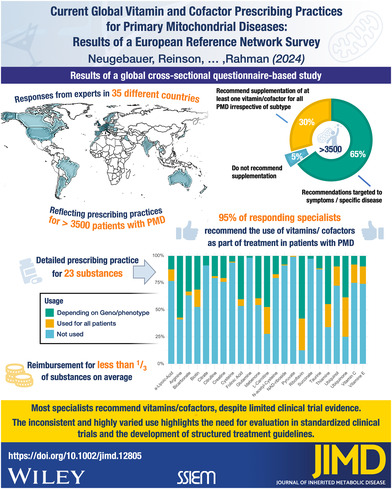

## 1 INTRODUCTION

Primary mitochondrial diseases (PMD) account for a group of almost 400 different genetically inherited disorders^1^ that result from dysfunction of the oxidative phosphorylation (OXPHOS) system or other essential mitochondrial functions due to pathogenic mutations in either mitochondrial (mtDNA) or nuclear DNA (nDNA). Although each individual disorder is rare, collectively they represent one of the largest groups of inherited metabolic disorders (IMD)^2^ with an estimated minimal prevalence of 1 in 4300 for PMD presenting in adults and children.^3^

Owing to the clinical heterogeneity and rarity of individual PMD, there are limited data from preclinical studies, clinical case reports, or rarely randomised clinical trials to guide specific or symptomatic treatment options, and most PMD continue to be without any curative or evidence‐based therapies.^4^

Vitamins are essential organic compounds that catalyse or function as cofactors in enzymatic reactions. They cannot be synthesised de novo in sufficient quantities by the human body and thus must be obtained from dietary sources.^5^ In the field of PMD, clinicians often prescribe one or more vitamin/cofactor(s). In the case of some specific disorders of vitamin transport or metabolism, or of coenzyme biosynthesis, the aim is to bypass the transporter or enzymatic defect. For other PMD, supplementation aims to reduce oxidative stress or toxic metabolites, augment cellular energy stores, compensate for a defect by bypassing defective reactions or providing an excessive supply of the cofactor, enhance electron transport chain function and ultimately alleviate the patient's health complaints and/or prevent further disease progression.^6^

Typical vitamins and cofactors used with this rationale include thiamine (vitamin B1), riboflavin (vitamin B2), biotin (vitamin B7), coenzyme Q_10_ (CoQ_10_: ubiquinone and its derivative ubiquinol) or idebenone, vitamin B3 (niacin, nicotinamide) and alpha lipoic acid. Other supplements include energy substrates such as L‐carnitine, creatine or anaplerotic substances such as citrate, succinate, and pyruvate. In addition, antioxidants including N‐acetylcysteine, vitamin E (α‐tocopherol and other vitamers), ascorbic acid (vitamin C), and nitric oxide (NO) precursors such as arginine or citrulline are frequently used in the context of PMD.^7^, ^8^ Some physicians prefer to prescribe combinations of these supplements as a “mitochondrial cocktail,” but this is not a universal practice. An important consideration is that there is currently no clinical trial evidence for the use of any of these supplements, either alone or in combination.^9^

While the general value of vitamin and cofactor supplementation in PMD is controversial and no clear advantage could be demonstrated in systematic reviews of existing clinical trials,^9^ many clinicians use supplementation as part of their treatment regimen^8^, ^10^, ^11^ with justification mainly based on efficacy in preclinical models,^12^ case reports or personal observations. Previous recommendations for nutritional supplements have been published from the North American Mitochondrial Disease Consortium, the Mitochondrial Medicine Society^8^, ^10^, ^13^ and individual centres in North America.^12^ In a subgroup of PMD (e.g., defects in cofactor metabolism or certain transporters^14^, ^15^), benefit of certain treatments in pharmacological doses has been demonstrated. However, the choice of treatment, combination of therapies and dose varies considerably among clinicians, further reflecting the multiplicity of agents, heterogeneity of the disease, and lack of controlled randomised trials in the field.^9^

To gain further insight into the current clinical practice of vitamin/cofactor supplementation among physicians treating children and adults affected by PMD, we conducted a worldwide cross‐sectional questionnaire study with the ultimate aim of laying the foundation for future research and development of recommendations and guidelines for their use in PMD. Although previous surveys frequently enquired about the use of vitamins or cofactors in general,^7^, ^8^, ^11^ to our knowledge there has been no data collection with a global perspective to date, and previous studies did not provide insight into which substances or combinations are used for specific conditions, a gap that we hope to close with this study.

## 2 METHODS

### 2.1 Questionnaire development and distribution

A web‐based questionnaire on the use of vitamins/cofactors was designed by members of the European Reference Network (ERN) for Rare Hereditary Metabolic Disorders (MetabERN), within the subnetwork responsible for pyruvate metabolism, mitochondrial oxidative phosphorylation disorders, Krebs cycle defects, and disorders of thiamine transport and metabolism [PM‐MD]. Several iterations of the draft survey were reviewed by members of the PM‐MD subnetwork and pre‐tested for clarity and comprehensiveness, before the final questionnaire was approved by all members of the subgroup. The survey was conducted online between August 16^th^, 2023 and January 31^st^, 2024 via a platform supported by the University of Tartu, Estonia.

The questionnaire consisted of 62 mostly multiple‐choice items addressing the specific use of different vitamins and cofactors, with a stepwise approach if clinicians specified that they use certain substances not for all patients with PMD but only in specific conditions. There was also the possibility to add free‐text comments.

The questionnaire was distributed to physicians treating patients with PMD through national metabolic societies, members of different ERN organisations (MetabERN, NMD‐ERN, EpiCare‐ERN, RND‐ERN, and Eye‐ERN, which together comprise the InterERN Mitochondrial Working Group^16^), patient organisations and global PMD specialists known to the members of the PM‐MD Network. Efforts to increase participation included follow‐up emails and reminders. The survey was available in English only.

An overview of the questionnaire and full list of queries is available in Appendix S1.

### 2.2 Data analysis

Data were pre‐screened for completeness and duplicates. Only complete, non‐duplicate questionnaires were included and analysed. Data entry was personalised to respondent and centre to control for multiple entries from the same individual. Data security and confidentiality were ensured by storing data on secure servers with restricted access. Only pseudonymised data were used for analysis to protect respondent identities. Due to the nature of the study, ethics committee approval was not required. Multiple entries from single centres were permitted to reflect real‐world treatment approaches.

Data were analysed with R (version 4.4) and IBM SPSS v.29.0 (SPSS Inc., Chicago, IL, USA) using descriptive statistics and grouping according to age group, number of patients treated as well as country of practice. Differences between country of practice, and number and age of patients treated were assessed using univariate analysis with Fisher's exact test and factor analysis with multiple correspondence analysis. Correlation between reimbursement and use was explored using Spearman's correlation coefficient. *p* ≤ 0.05 was considered statistically significant.

## 3 RESULTS

### 3.1 Responses and participant characteristics

**FIGURE 1:**
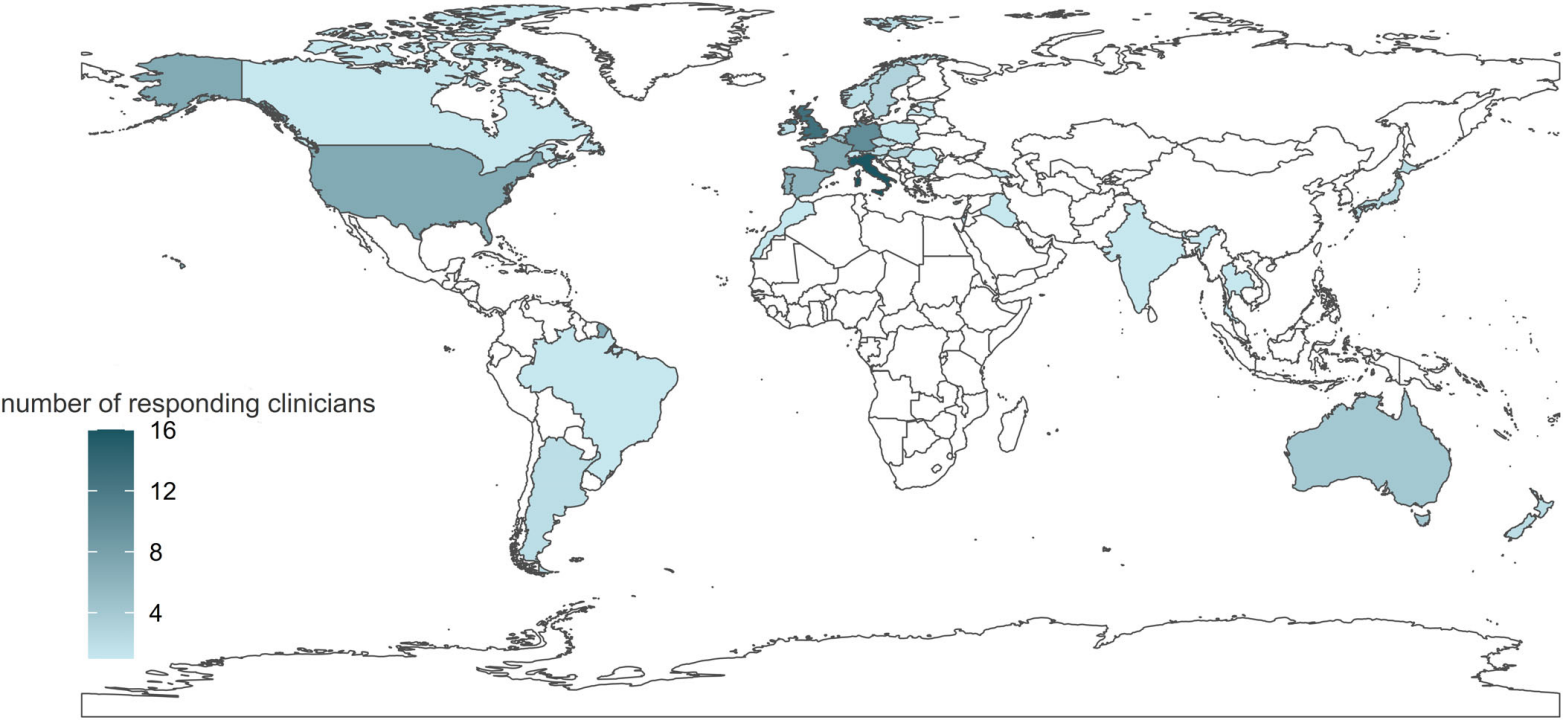
World map displaying the geographic distribution of responding clinicians and the number of responses from each country. In total, 112 respondents from 35 countries contributed their experience. The majority of respondents are from Europe (78%), while areas with potentially substantial incidence such as Asia and South America are underrepresented, and there were no respondents from Sub‐Saharan Africa.

**FIGURE 2:**
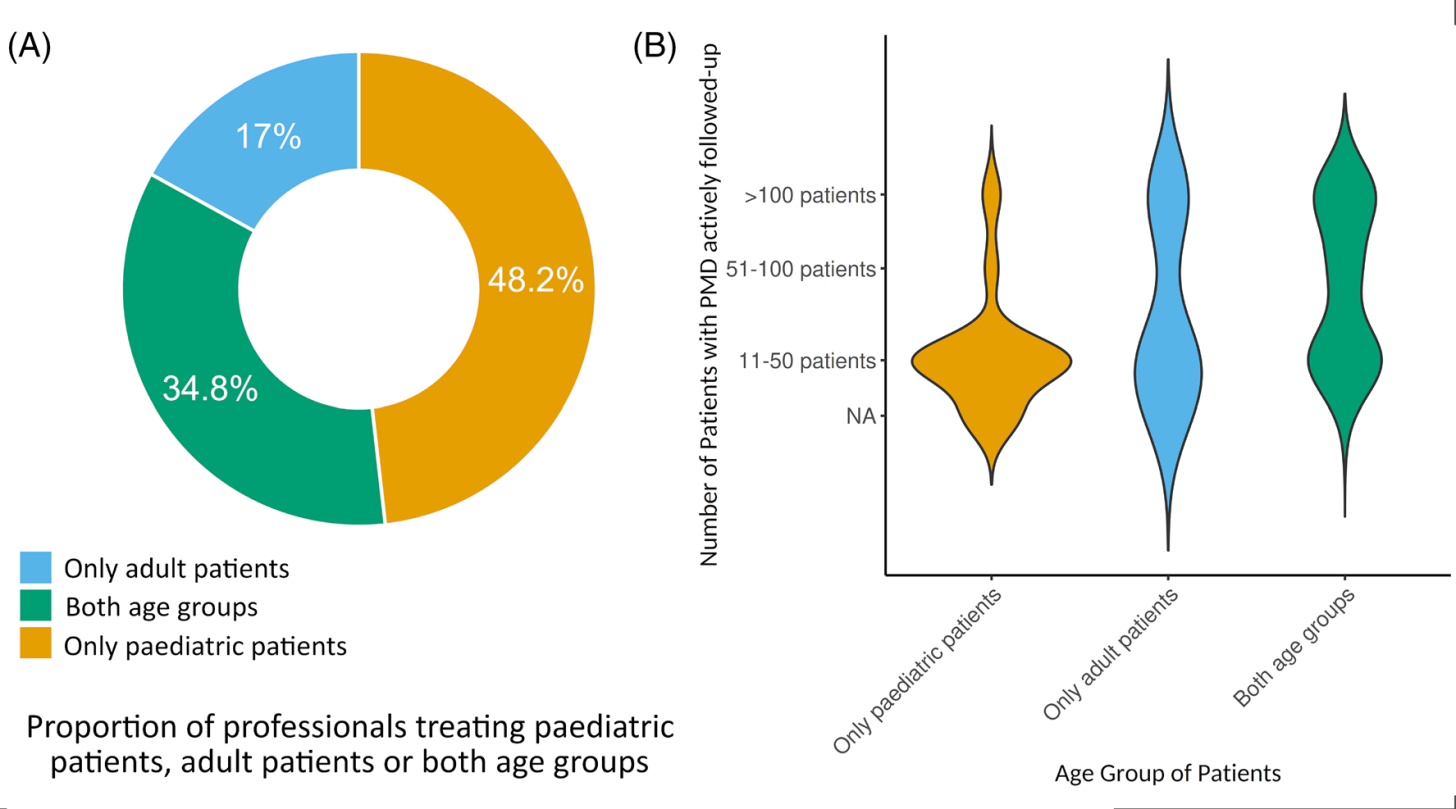
(A) Proportion of professionals treating paediatric patients with PMD, adult patients or both age groups. This figure provides an overview of the proportion of professionals that exclusively follow paediatric patients with PMD, exclusively follow adult patients, or care for both age groups. In contrast to previous studies, this survey received a notably higher response rate from paediatricians, with 48.2% of respondents only managing patients under 18 years old, and 17% managing both paediatric and adult patients with PMD. (B) Distribution of actively followed PMD patients between clinicians treating only paediatric patients, only adult patients or both age groups. This figure illustrates the distribution of patients with PMD actively followed by each respondent. Notably, 21.4% of respondents manage more than 100 patients with PMD. Most paediatricians follow between 11 and 50 patients with genetically confirmed PMD. While the majority of respondents are paediatricians, most clinicians who follow more than 100 patients primarily treat adult patients (83%, 19/23) (*p* = 0.003), either alongside paediatric patients or exclusively.

A total of 112 respondents from 35 different countries and six different continents (Figure 1) shared their experience, with 48.2% (54/112) involved only in the care of patients <18 years and 51.8% (58/112) additionally (34.8%, 39/112) or solely (17%, 19/112) responsible for adult patients (Figure 2). Together, they account for a minimum of actively followed patients with PMD of at least 3500 patients. Most respondents were paediatricians, specialising in IMD (49%, 55/112) or paediatric neurology (25%, 28/112). Twenty‐one clinicians (19%, 21/112) were adult neurologists, while only a few respondents worked in internal medicine (2/112) or adult metabolic medicine (1/112). Five percent were geneticists (6/112) and one person was mainly involved in diagnostics. Thirty‐five percent of paediatricians (28/81) were also following patients above 18 years old, and six adult neurologists also followed children.

The majority of clinicians actively follow between 11 and 50 patients with genetically proven PMD (53.6%, 60/112); 17 clinicians (15.2%) follow fewer than 10 cases; and 11 (9.8%) between 51 and 100 cases. However, a subset of 21.4% (24/112) actively care for >100 patients with PMD. In contrast to the majority of respondents being paediatricians, most of the clinicians following >100 patients primarily treat adult patients (83%, 19/23) (*p* = 0.003), either in addition to paediatric patients or exclusively (21%, 5/23) (Figure 2B).

We observed geographical differences in the subgroup of specialists following >10 cases (*p* = 0.03). Specialists located in North America (87.5%, 7/8) are more likely to follow more than 100 cases compared to those from other regions, especially European countries such as the United Kingdom (31%, 4/13), Hungary (33%, 1/3), France (28%, 2/7), Italy (25%, 4/16), Germany (25%, 2/8), and Spain (0%, 0/5).

### 3.2 Use of vitamins and cofactors

The vast majority (95%, 106/112) of clinicians incorporate the use of vitamins or cofactors in the treatment of PMD; a small percentage (5%, 6/112) abstain from their use entirely. Of the latter group, 67% (4/6) actively follow 11–50 patients with PMD, but one specialist who does not use vitamins/cofactors cares for >100 patients.

**FIGURE 3:**
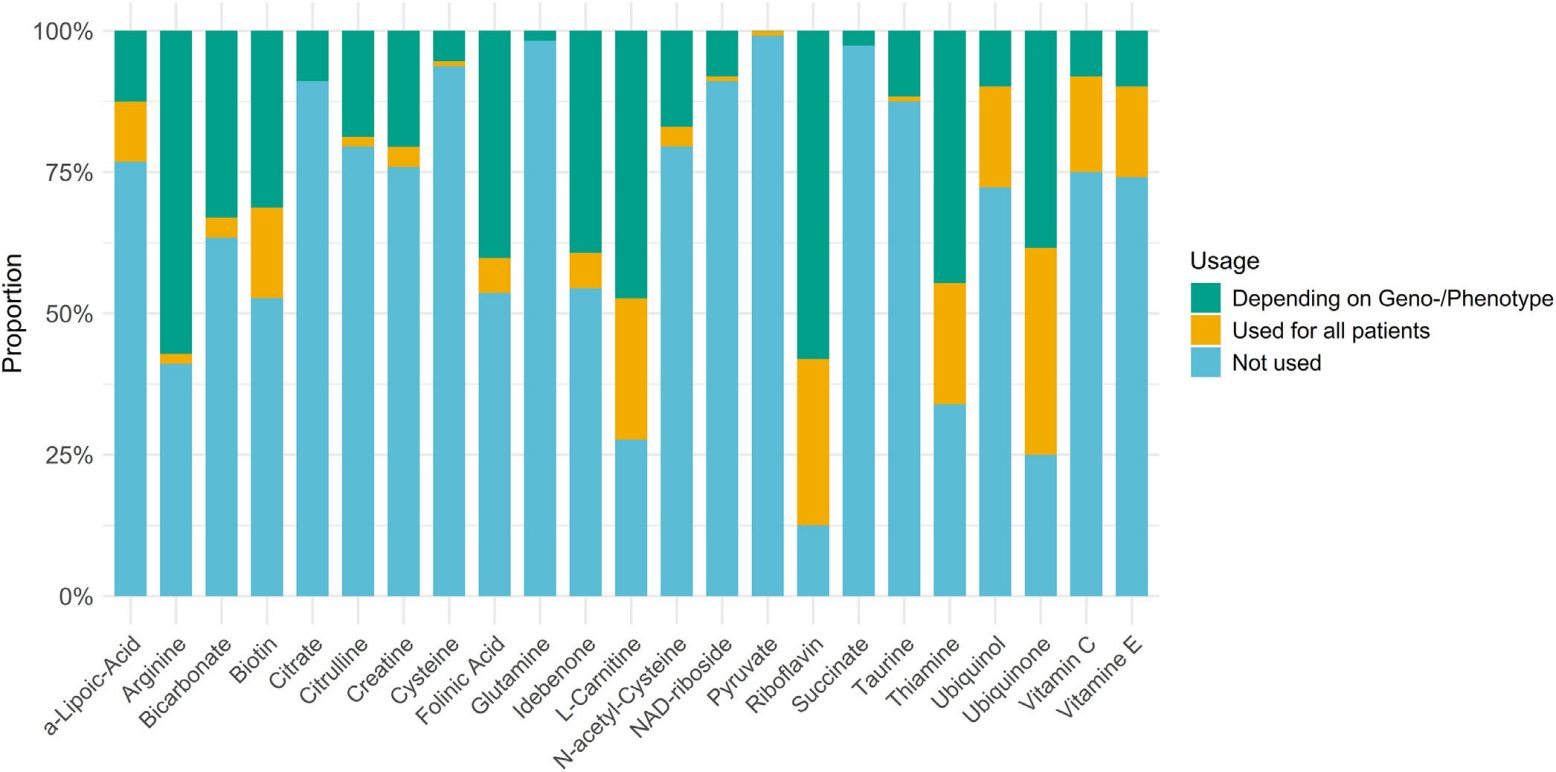
Prescribing differences for the various vitamins and cofactors. This figure displays the prescribing practices for each vitamin/cofactor addressed in the survey, indicating the proportion of professionals that use the specific vitamin or cofactor universally for every patient with PMD irrespective of the geno‐ or phenotype, as well as those who prescribe based on specific geno‐ or phenotypes.

The five vitamins/cofactors most frequently reported to be used, are ubiquinone (77.6%, 87/112), riboflavin (87.5%, 98/112), L‐carnitine (72%, 81/112), thiamine (66%, 74/112), and ubiquinol (27.6%, 31/112) (Figure 3). We found that the use of glutamine (1.8%, 2/112), succinate (2.6%, 3/112) and pyruvate (<1%, 1/112) was extremely limited in the treatment of PMD.

Riboflavin was the vitamin most often prescribed in the treatment of PMD, with 87.5% (98/112) of clinicians confirming its use either irrespective of geno‐/phenotype or as a targeted treatment for specific cases. Among these, 33.7% (33/98) administer riboflavin to all patients with a genetically proven PMD, while others reserve its use for particular geno‐/phenotypes. Apart from conditions with established efficacy^10^, ^11^ such as defects of riboflavin transport or intracellular metabolism (FAD synthase (FLAD1) deficiency), multiple acyl‐CoA dehydrogenase deficiency (MADD, caused by deficiency of ETFDH, ETFA, or ETFB), and certain complex I defects (e.g., deficiency of ACAD9, or the complex I subunits NDUFV1 and NDUFV2^17^), approaching half of the surveyed clinicians prescribe riboflavin to patients with other complex I (50%, 56/112) or complex II (41%, 46/112) deficiencies, as well as in cases of congenital lactic acidosis (49%, 55/112), suspected or confirmed mitochondrial cardiomyopathy (46%, 51/112), and primary mitochondrial myopathy (43%, 48/112). Similar numbers arise if one accounts for expertise. Riboflavin is prescribed by 87% (20/23) of clinicians with high expertise (as they care for >100 PMD patients each), with 39% (9/23) stating that they recommend its use for all patients regardless of the geno‐/phenotype while 47% (11/23) reserve its use for specific conditions.

Ubiquinone is recommended by 77.6% (87/112) of clinicians for the treatment of PMD, either for a specific geno‐/phenotype or universally in all patients with PMD. While 36.6% (41/112) of clinicians prescribe ubiquinone irrespective of geno‐/phenotype, a subset of 5% (6/112) stated that they would perform a trial of ubiquinone for several months and continue based on symptomatic improvement. Apart from its established role in different forms of CoQ_10_ biosynthesis defects (with high dose supplementation),^10^ clinicians were most likely to prescribe ubiquinone in the treatment of Kearns–Sayre syndrome (50%, 56/112), chronic treatment of mitochondrial encephalopathy, lactic acidosis, and stroke (MELAS) (49%, 55/112) or Leigh syndrome spectrum (47%, 53/112), or when confronted with a patient with mitochondrial cardiomyopathy (47%, 53/112) or primary mitochondrial myopathy (54%, 61/112).

Ubiquinol, the reduced form of oxidised ubiquinone, was noticeably less frequently used than ubiquinone—but also not consistently available in every country. Overall, 28% (31/112) of specialists recommend the use of ubiquinol, with most of them (64.5%, 20/31) administering it to all patients with PMD regardless of their specific condition or phenotype. There is no clear preference for its use in a particular disease or phenotype. In most conditions a mean of 21% of clinicians opt for the use of ubiquinol.

Among clinicians with significant experience, defined as treating over 100 PMD patients, 87% (20/23) recommend the use of CoQ_10_ (ubiquinone or ubiquinol).

The use of L‐carnitine is recommended by 72% (81/112) of clinicians. Of these, almost two‐thirds (64%, 52/81) use it only for specific conditions or phenotypes, while the rest use it in all patients with PMD. Specialists managing more than 100 patients recommend L‐carnitine less frequently, with only 52% (12/23) advocating for its use. Of those, two thirds (66%, 8/12) restrict its use to particular genotypes or specific symptoms. Its use is particularly favoured for myopathic phenotypes, with 41% (46/112) of clinicians using it for primary mitochondrial myopathy and 29% (32/112) for benign reversible mitochondrial myopathy. Thirty‐six percent (40/112) of clinicians prescribe L‐carnitine in suspected or proven mitochondrial cardiomyopathy, 31% (35/112) during acute decompensation in Leigh syndrome and 32% (36/112) in acute liver failure of unknown cause in neonates. Some clinicians (19%, 21/112) base their decision to use L‐carnitine on plasma carnitine concentration, administering it only in cases with biochemically confirmed deficiency.

The use of thiamine is advocated by two thirds of overall clinicians (66%, 74/112) and by 60% (14/23) of specialists with extensive expertise (managing more than 100 patients). Although 21% (24/112) of clinicians prescribe thiamine irrespective of geno‐/phenotype, most tend to recommend it only for specific conditions, particularly pyruvate dehydrogenase complex deficiency (55%, 62/112), neonatal lactic acidosis (44%, 49/112) or acute initial presentations or decompensations of a patient with Leigh syndrome spectrum (41%, 46/112).

### 3.3 Treatment of specific diseases with vitamins or cofactors

Certain vitamins/cofactors are used more frequently in the treatment of specific diseases. The use of nitric oxide precursors L‐arginine and L‐citrulline is significantly higher in the treatment of MELAS syndrome compared to other PMD (*p* < 0.001). More than half of clinicians (54%, 60/112) use L‐arginine and 13% (15/112) L‐citrulline in the setting of acute stroke‐like episodes in suspected or confirmed MELAS syndrome. Most of them continue to use these substances in the chronic treatment of patients with MELAS syndrome; 42% (48/112) use L‐arginine and 15% (17/112) use L‐citrulline. Notably, L‐citrulline is used by all clinicians based in the US and Canada, while use in other countries is more heterogeneous. Ten percent state that they use additional taurine in chronic treatment of MELAS.

L‐arginine use during acute stroke‐like episodes in suspected or confirmed MELAS syndrome was promoted by 50% of specialists with extensive expertise (12/24). The use of L‐citrulline in acute or chronic treatment of MELAS syndrome is notably more common among these highly experienced specialists compared to clinicians overall, with 33% (8/24) endorsing its use in this condition.

Folinic acid is rarely used in PMD (<10% for most PMD), except in the context of measured low 5‐methyltetrahydrofolate (5‐MTHF) levels in cerebrospinal fluid. However, 31% (35/112) of clinicians use folinic acid in Kearns–Sayre syndrome, particularly if there is evidence of cerebral white matter disease (as stated in free‐text comments), and 18% (20/112) use it in Pearson syndrome, another single large‐scale mtDNA deletion syndrome.

Idebenone is used by 43 clinicians (38%, 43/112) in the treatment of mitochondrial optic neuropathy (i.e., Leber hereditary optic neuropathy, LHON; there is, however, limited region‐specific approval).

N‐acetylcysteine, although not widely used for most PMD, is used more frequently in TRMU deficiency and ethylmalonic encephalopathy (the latter was not specifically included in the questionnaire but mentioned by several clinicians in free‐text comments). There were similar findings for vitamin B3 (niacin or nicotinamide riboside), which was preferred by some respondents for patients with mitochondrial myopathy, NAD(P)HX dehydratase (NAXD) and NAD(P)HX epimerase (NAXE) deficiency (not specifically included in the survey but mentioned by some respondents in free‐text comments).

### 3.4 Treatment of patients with suspected PMD

Since most clinicians stated that they prescribe vitamins or cofactors depending on the specific geno‐/phenotype, we explored the use of different vitamins/cofactors in some common presentations of PMD.

In the case of neonatal lactic acidosis, most clinicians stated that they prescribe riboflavin (51%, 57/112), ubiquinone (46%, 52/112), thiamine (44%, 49/112), biotin (33%, 37/112) and L‐carnitine (30%, 34/112), or in many cases, a combination thereof. Bicarbonate was preferred by 33% (37/112) of clinicians as a buffer therapy, while only 4.4% (5/112) opt for the use of sodium citrate in cases of neonatal lactic acidosis. With an overlapping phenotype, clinicians commonly opt for similar compounds in the treatment of acute initial decompensation in Leigh syndrome spectrum disorder, with 46% (51/112) preferring to use ubiquinone, 44% (49/112) riboflavin, 38% (42/112) thiamine, 38% (42/112) biotin and 31% (35/112) using L‐carnitine in this setting. Faced with a suspected or confirmed mtDNA depletion syndrome, almost half of clinicians use ubiquinone (45%, 50/112), fewer use riboflavin (37%, 41/112) and thiamine (23%, 26/112), and there was substantially less use of L‐carnitine (13%, 14/112) compared to congenital lactic acidosis or Leigh syndrome spectrum. In patients with cardiomyopathy associated with suspected or confirmed PMD, 54% (60/112) of specialists recommend CoQ_10_ (47% (53/112) opt for ubiquinone, 21% (23/112) for ubiquinol), 48% (54/112) use riboflavin, 37% (41/112) use L‐carnitine and 29% (33/112) thiamine.

### 3.5 Comparison of substance use between specialties and treated age categories

There is a significant difference in both the frequency and assortment of vitamin/cofactor usage among clinicians exclusively managing adult patients versus those treating paediatric patients (*p* < 0.001). Significant divergent patterns emerge in the use of individual vitamins/cofactors across these professional groups. In particular, the use of biotin (*p* < 0.001), thiamine (*p* ≤ 0.001), taurine (*p* < 0.001), alpha‐lipoic acid (*p* = 0.037) and bicarbonate (*p* = 0.04) is more prevalent among paediatricians compared to those working in adult medicine. In contrast, idebenone is more commonly used in adult medicine.

### 3.6 Regional differences in the use of vitamins and cofactors

**FIGURE 4:**
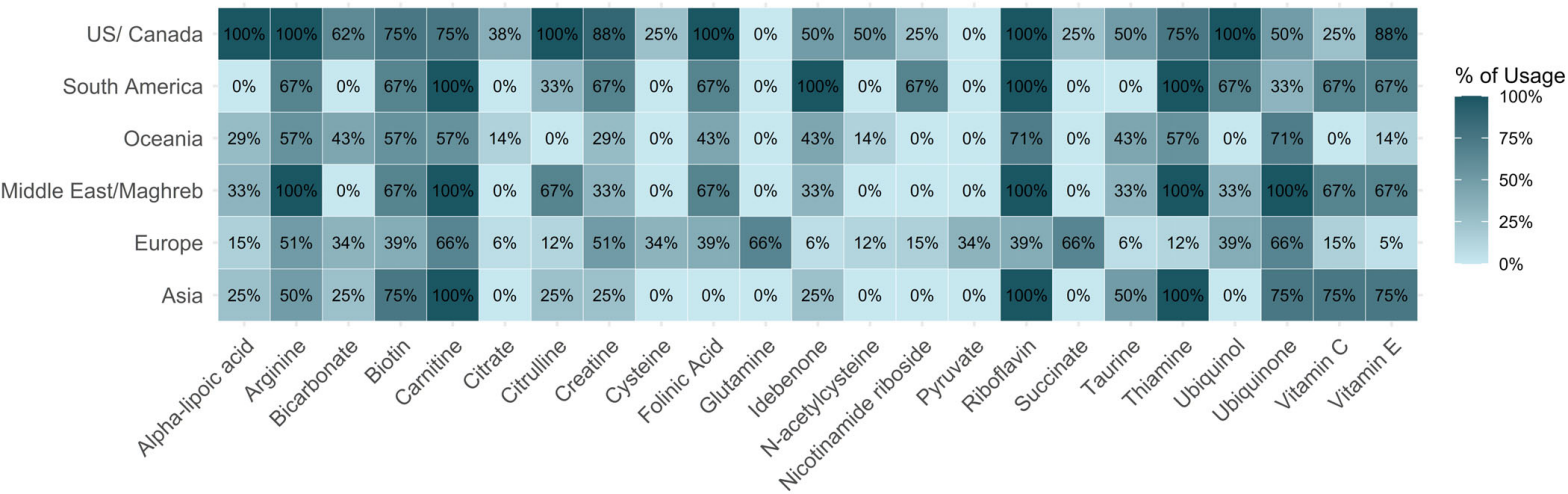
Regional differences in vitamin/cofactor use. This colour‐enhanced table illustrates the variation in the use or prescription of various vitamins and cofactors by professionals across different regions. Each row corresponds to a specific geographic region, listing the proportion of professionals in each region who report prescription of this vitamin or cofactor.

**FIGURE 5:**
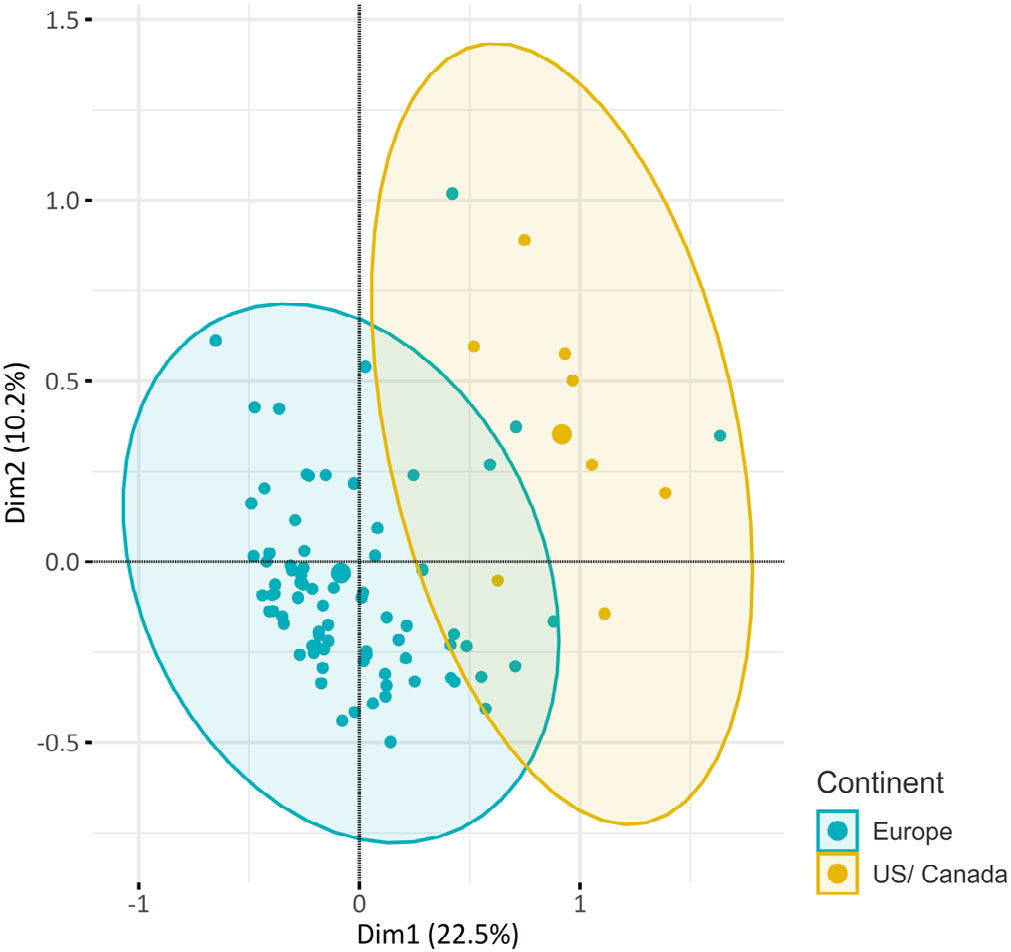
Association between geographic location (North America vs. Europe) of specialists and prescribing practices of vitamins/cofactors. Multiple correspondence analysis (MCA) was utilised to investigate the association between the geographic location (North America vs. Europe) of specialists and their prescribing practices for various vitamins and cofactors. Each vitamin and cofactor is treated as a categorical variable, with responses categorised into whether or not a substance is used, and whether its use is universal or specific for certain geno‐/phenotypes. The MCA revealed a distinct pattern between specialists from Europe and North America regarding the spectrum of substances and their targeted application in specific diseases. An MCA plot involving the other regions can be found in Appendix S1.

Multiple correspondence analysis exploring the association between geographical location of specialists and their use of various vitamins/cofactors revealed a distinct pattern between specialists from Europe and North America regarding the spectrum of substances and their targeted application in specific diseases (Figures 5 and S2). In the subgroup of specialists that are following >10 cases we observed significant differences between the countries of residence (*p* = 0.03) (Figure 4, regional differences in vitamin/cofactor use, responses of all specialists). Ubiquinol, while used by North American clinicians for every PMD patient regardless of the geno‐/phenotype, is not used in Australia/New Zealand and is only used by 21.5% of clinicians in Europe (*p* < 0.001), where use of ubiquinone is favoured (Europe 76% vs. US 50% vs. Oceania 72%). Vitamin C is recommended in all patients regardless of genotype by 75% of responding specialists from Asia and 66% of clinicians in South America but not used at all by responding clinicians from Australia/New Zealand, while 12.5% of clinicians from North America and 20% of clinicians from Europe recommend its use in certain cases. L‐citrulline in addition to L‐arginine in MELAS syndrome is mostly used in North America (100% of US clinicians, 11.8% of Europeans, *p* < 0.001). Alpha‐lipoic acid is used by every North American clinician, but only 15% (14/88) of European clinicians (*p* < 0.001) in the treatment of PMD. Vitamin E is used significantly more often in North America than in Europe (*p* < 0.001) with 87.5% (7/8) of North American clinicians prescribing it compared to 15% (14/88) in Europe.

**FIGURE 6:**
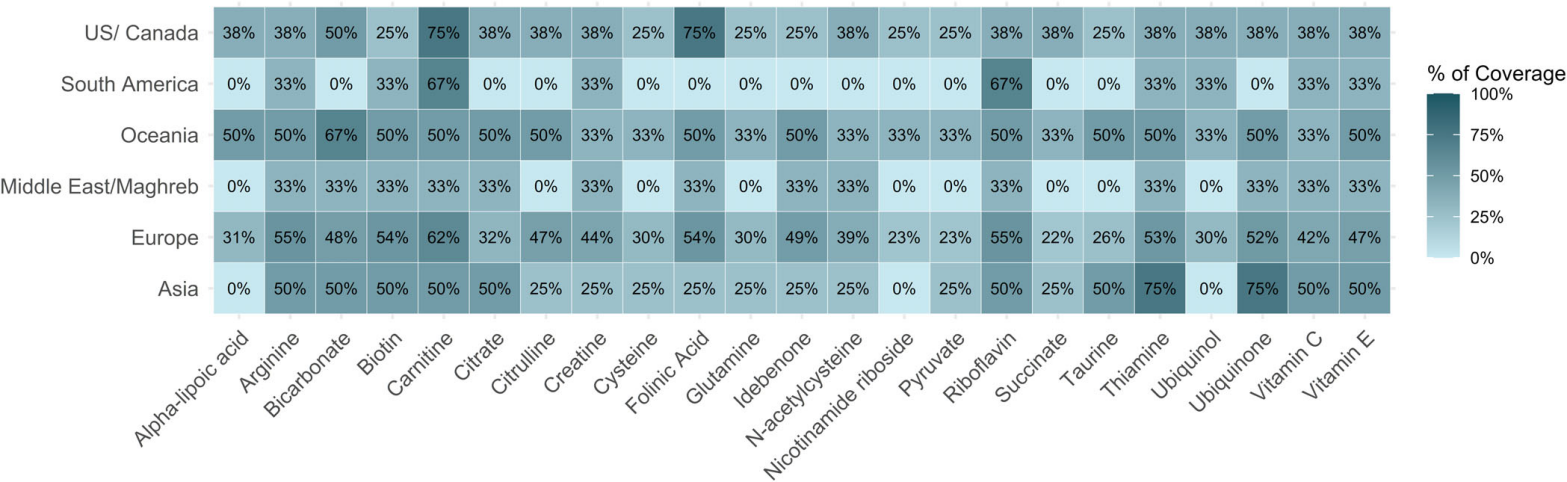
Regional differences in reimbursement rates for the different vitamins and cofactors. Regional disparities in reimbursement rates for the different vitamins and cofactors are depicted in this colour‐enhanced table. Each row represents a specific geographic region and indicates the proportion of professionals who reported reimbursement for each respective vitamin or cofactor in their region.

### 3.7 Global reimbursement trends for vitamins and cofactors

We analysed the correlation between use and reimbursement of vitamins and cofactors globally. Overall, reimbursement rates are low for most vitamins/cofactors. For most of these substances, insurance schemes do not cover the costs or only provide reimbursement following an individual request on a case‐by‐case basis (according to individual statements). While there are regional differences in cost compensation for the different supplements (Figure 6)‐ with Europe and Australia/New Zealand having a higher rate of overall coverage compared to North and South America, there does not appear to be a significant correlation between use of most vitamins/cofactors and cost coverage. An exception is ubiquinol, for which there is a moderately strong but not statistically significant correlation between use and reimbursement (Spearman correlation, rho = 0.63, *p* = 0.18).

### 3.8 Availability of compounds in different countries

In general, availability appears to be sufficient for most vitamins/cofactors in most countries (mean availability of a compound = 77% ± 17%) (Figure S1). Ubiquinol is only available for 59.8% (67/112) of specialists. Nicotinamide riboside appears to have limited availability, with only 51.8% (58/112) of specialists being able to access it. Availability of idebenone is limited for 37.5% (3/8) of specialists from North America and across many European countries (not available for 24% (22/89) of specialists from Europe).

## 4 DISCUSSION

We present the first global cross‐sectional questionnaire‐based study aiming to characterise the current clinical practice of vitamin/cofactor supplementation among physicians treating children and adults affected by PMD. Despite their collectively high frequency,^2^, ^3^ PMD are among the most heterogeneous subtypes of IMD, resulting in a lack of therapeutic options for affected patients^4^ and necessitating a predominantly symptomatic treatment approach. The questionnaire collected information on the recommendations for vitamin/cofactor use by different specialists in the field of PMD, with a high rate of responses from clinicians with notable expertise in this field; 21% of respondents each care for >100 patients with PMD. Compared to previous studies, this survey had a significantly higher rate of responses from paediatricians.^11^, ^13^

This study allows us to draw a number of important conclusions.

### 4.1 Wide use of vitamins and cofactors in the treatment of PMD

Vitamins and cofactors are used by the vast majority of specialists (95%, 106/112) as a treatment modality in PMD. This contrasts with existing evidence that does not show clinical trial‐based evidence for most PMD. Several potential reasons may explain this discrepancy.

The widespread use might be a result of the absence of alternative effective treatments, compelling clinicians to rely on vitamins and cofactors. It is also possible that both patients and clinicians may observe minor effects or benefits (maybe only in subgroups of patients), justifying continued use despite the lack of robust evidence. This approach underscores the pragmatic nature of current clinical practices, where clinicians often initiate treatment based on empirical observations and symptomatic improvement rather than conclusive genetic confirmation of the underlying diagnosis.^18^ A contributing factor here is the lack of reliable biomarkers and objective outcome measures to assess the efficacy of these treatments in this clinically, biochemically, and genetically heterogeneous group of disorders,^4^, ^19^ leading to continued use of vitamins/cofactors based on subjective observations. Furthermore, vitamins and cofactors are often used as an initial treatment strategy when faced with a not genetically confirmed but suspected PMD, to address the potentially treatable subgroups within PMD. Alternatively, the use of certain substrates might stem from theoretical concepts and is aimed at logically counteracting specific pathomechanisms such as NO depletion, oxidative stress, or at providing a chaperone‐like effect.

A further reason for the discrepancy could be that vitamins/cofactors are generally considered to be “harmless” therapies. While this is probably true for most vitamins/cofactors, and most considered indications, there are exceptions where their use has the potential to cause significant harm even at recommended doses. For instance, L‐carnitine can be exceptionally beneficial for a child with dilated cardiomyopathy caused by primary carnitine deficiency (carnitine transporter deficiency) but may cause life‐threatening events or worsening of underlying condition in other diseases with cardiomyopathy such as TANGO2 deficiency,^20^ and is suspected to increase the accumulation of lipotoxic long‐chain acylcarnitines and thus the risk of cardiac arrhythmias and rhabdomyolysis in long‐chain fatty acid oxidation disorders.^21^, ^22^ Additionally, high‐dose and long‐term vitamin C supplementation can lead to oxalate nephropathy.^23^ Supplementation with biotin can lead to interference with avidin‐based immunoassays (specifically relevant for thyroid function tests), potentially leading to additional unnecessary investigations or even treatment changes in PMD patients where thyroid impairment is common.^24^ Thus, it is our belief that agnostic approaches of vitamin/cofactor supplementation should be carefully weighed for their potential harm and benefit.

### 4.2 Clear evidence of targeted vitamin or cofactor supplementation in certain diseases

Our study provides clear evidence that clinicians use targeted supplementation in diseases where preclinical experiments, single case reports or natural history studies have shown supportive findings. Since 84% (94/112) of clinicians base their treatment recommendations on personal reading or experience, the treatment of certain diseases, where evidence from clinical or preclinical studies supports the use of specific vitamins or cofactors, differs substantially. It is noteworthy that clinical practice guidelines providing evidence‐based recommendations on the use of vitamins and cofactors in PMD are scarce. Often, recommendations are local and dependent on institutional or national practices.^16^ Consequently, certain vitamins/cofactors are used more frequently in the treatment of specific diseases compared to others. This was particularly evident in the treatment with L‐arginine and L‐citrulline of acute stroke‐like episodes in MELAS syndrome^25^, ^26^ and, to a lesser extent, taurine supplementation in chronic MELAS treatment.^27^ The use of L‐arginine is under debate, as a systematic review conferred no demonstrable benefit in acute or prophylactic treatment of MELAS.^28^ The increased use of folinic acid in Kearns–Sayre syndrome, N‐acetylcysteine in ethylmalonic encephalopathy and TRMU deficiency, or idebenone in LHON, aligns with variable levels of existing evidence for their benefit in these conditions.^29^, ^30^, ^31^, ^32^, ^33^

A similar approach was observed in cases where a PMD was suspected, and the phenotype matched one of the treatable conditions. Treatment strategies often involved thiamine, CoQ_10_, biotin, and/or riboflavin and thus addressed potentially treatable diseases involving cofactor metabolism or transporters. For instance, riboflavin was used more often in cardiomyopathy, targeting potential deficiency of ACAD9, FAD synthase or multiple acyl‐CoA dehydrogenase. The questionnaire did not include vitamin B5 (pantothenate), which has recently been suggested as a treatment for some disorders of CoA biosynthesis (e.g., phosphopantothenoylcysteine synthetase (PPCS) deficiency^34^) and together with other B vitamins for TANGO2 deficiency, a PMD mimic with potentially lethal cardiac manifestations.^35^, ^36^ Biotin and/or thiamine were used more frequently in neonatal lactic acidosis or acute Leigh syndrome, to treat the possibility of deficiency of the SLC19A3 thiamine transporter, biotinidase or pyruvate dehydrogenase complex.^37^ In particular, for patients with SLC19A3 thiamine transporter deficiency early initiation of thiamine and biotin can dramatically improve neurological outcome and significantly impact survival.^38^, ^39^ Particular attention should also be paid to specific pathogenic variants that may be responsive to thiamine or riboflavin.^37^, ^40^, ^41^

Some clinicians indicated that they would later discontinue treatment if these diagnoses were excluded. Given the restrictive reimbursement in many countries, clear consensus recommendations could promote insurance coverage for treatment of these specific diseases.

### 4.3 Prescribing differences related to clinical specialty and region

This study reveals significant regional and specialty related differences in PMD treatment.

Adult physicians or specialists who treat both paediatric and adult patients are more likely to manage greater than 100 patients with PMD compared to paediatricians (Figure 2B). This distribution is certainly influenced by the longer time span of adult life compared to childhood, but also likely reflects the higher prevalence of PMD manifesting in adults and the historically greater number of paediatricians specialising in IMD, leading to a broader dispersal of paediatric patients.

Vitamins and cofactors are more likely to be used in paediatric than adult cohorts, with significant differences in the use of individual vitamins/cofactors. This observation is of particular importance. Historically, no or only small numbers of paediatric patients were involved in randomised clinical trials. Extrapolation of data obtained from adult clinical trials is potentially inappropriate and neglects this distinct patient cohort that often has a more severe phenotype or subtypes of PMD that are underrepresented in an adult cohort. Thus, data obtained in previous clinical trials might not capture partial or long‐term efficacy in paediatric patients.

Idebenone is prescribed more frequently in adult medicine, reflecting its use primarily in LHON, where disease onset is typically in young adulthood.^42^ It has gained approval from the European Medicines Agency (EMA), the Therapeutic Goods Administration (TGA) Australia (although not subsidised)^43^ and the Japanese government for treating LHON but has not received approval by the US Food and Drug Administration (FDA).^33^ However, in the US and several other countries, idebenone is available as a dietary supplement, although access appears to be restricted, as indicated by 37.5% of US specialists in this survey. Despite being approved by the EMA, questionnaire responses imply that idebenone availability is limited across many European countries.

Niacin‐based therapies can be life‐saving in the rare NAXD and NAXE deficiencies.^44^ There is also preclinical evidence for enhancing mitochondrial biogenesis and potential improvement of mitochondrial myopathy.^45^ Nicotinamide riboside appears to be accessible to only half (51.8%) of clinicians.

There were substantial regional differences regarding the spectrum of substances used and their targeted application in specific diseases or phenotypes, particularly between specialists from Europe and those in North America. Although non‐European respondents were relatively under‐represented, suggesting a high likelihood of sampling bias, we could observe a higher tendency for a generalised approach (“mitochondrial cocktail”) among respondents based in North America compared to Europe. On the other hand, respondents from North America were more likely to treat large (>100) cohorts of patients with PMD than physicians from other regions, especially European countries. This may be due to a study bias as there were more responses from medium‐sized centres in central Europe which are more represented in MetabERN (which endorsed this project), but it could also reflect the differences in health networks and organisational structures of these countries.

While these observed differences clearly highlight the need for international recommendations, they also present an opportunity for (retrospective) comparative studies on treatment outcomes for specific diseases between these regions. The variability in treatment approaches among specialists underscores the need for standardised clinical guidelines that accommodate regional disparities and different clinical specialties.

### 4.4 Low rates of reimbursement and coverage of associated costs

In general, the reimbursement rates for vitamin/cofactor supplementation are low, even for widely used substances such as riboflavin, thiamine and CoQ_10_ (ubiquinone or ubiquinol). The possibility that economic considerations substantially influence the prescription of substances that were studied here cannot be excluded. This is particularly noteworthy for vitamin/cofactors with limited or region‐specific approval.

The preference of ubiquinol over ubiquinone is of high controversy among experts within the field of PMD. Both ubiquinone as well as its reduced derivative ubiquinol are highly hydrophobic substances with an inherently low bioavailability of oral supplements. The prevalent and often promoted idea that ubiquinol is superior in intestinal absorption, and thus more effective, lacks substantial support from biochemical and bioavailability studies,^46^, ^47^ where similarly low absorption rates of both substances and internal continuous interconversion have been demonstrated. Given that ubiquinol is on average approximately twice as expensive as ubiquinone and insurance coverage for ubiquinol is even more limited, the advisability of recommending ubiquinol for patients should be subjected to scrutiny.

Low reimbursement rates for vitamin/cofactor supplementation in general imposes an additional, and at times substantial, financial burden on families. Moreover, disparities in reimbursement rates highlight inequities in access to potentially beneficial treatments across different healthcare systems and regions. Consequently, there is a high need for further focused research and clinical trials examining the use of selected vitamins/cofactors in specific PMD or evidence from preclinical trials in appropriate disease model systems. Although this study provides important preliminary insights into expert‐based opinions regarding the use of vitamins/cofactors in PMD, more rigorous investigations or clear consensus statements are needed.

Our study has some limitations which need to be acknowledged. Differences in the definition and diagnosis of PMD among specialists may have affected the responses and comparability of the data. The use of vitamins/cofactors in specific diseases is likely skewed since there was no initial question about the spectrum of diseases treated by each respondent. This likely leads to an underestimate of the use of individual substances in certain diseases so the interpretation of these exact numbers should be approached with caution. On the other hand, the survey might have attracted participants who are already predisposed to using vitamins/cofactors, potentially skewing the results towards higher prevalence of use. Although this was a worldwide survey, respondents from Europe are overrepresented (78% of respondents), while areas with likely substantial incidence such as Asia and South America were underrepresented. This might reflect the endorsement of the survey by MetabERN, an EU initiative, and the additional spread through personal contacts of the authors but it also aligns with the insufficient representation of certain groups in biomedical research.^48^, ^49^ The survey did not include a question specifically addressing the duration limit of a treatment. Nevertheless, several clinicians expressed their intention to carry out time‐limited trials of certain vitamins/cofactors, continuing their use only if a benefit is observed. Respondents answered individually and subjectively to items, and data regarding cost coverage and availability might not accurately reflect the situation in their countries. Variations in availability and reimbursement of vitamins/cofactors across different regions might have influenced specialists' treatment decisions, biasing the results towards practices in areas with better access to these compounds.

## 5 CONCLUSION

In this study, we provide an overview of worldwide practices of vitamin and cofactor supplementation for the treatment of PMD. While limited in terms of representation of specific regions and the subjective nature of the responses, the results provide a detailed characterisation of current use across age groups and specialties. The inconsistent and highly varied use of vitamins and cofactors, coupled with the lack of robust clinical trial evidence and objective outcome measures, highlights the need both for standardised evaluation in randomised clinical trials and for the development of structured treatment recommendations and guidelines to improve outcomes for patients with PMD.

## AUTHOR CONTRIBUTIONS

JN: Conceptualising the content and the structure, formal analysis and data curation, drafting of manuscript, and conceptualization and creation of figures. KR: Conceptualising the content and the structure of the questionnaire, data curation and project administration. MB: Conceptualising the content and the structure of the questionnaire, formal analysis, critical revision of the manuscript. JHP, OH, EB and MS: Conceptualising the content, review and editing of the manuscript and figures. SR: Conceptualising the content and structure of the questionnaire and manuscript, supervision, drafting and editing of the manuscript, conceptualization and editing of figures. All authors: Critical revision of the questionnaire and manuscript.

## FUNDING INFORMATION

S.R. acknowledges grant funding from Great Ormond Street Hospital Children's Charity, the Lily Foundation and the National Institute of Health Research (NIHR) Great Ormond Street Hospital Biomedical Research Centre. K.R. acknowledges funding from the Estonian Research Council grant PRG471 and Estonian Research Council grant PRG2040. J.H.P. is an Else Kröner Memorial Stipend. The other authors did not receive financial support relevant to this study. The authors confirm that the views expressed are those of the authors. The content of the article has not been influenced by the funders.

## CONFLICT OF INTEREST STATEMENT

Shamima Rahman declares that she is an editor‐in‐chief of the Journal of Inherited Metabolic Disease and has provided consultancy on primary mitochondrial diseases to pharmaceutical companies as listed in the IJCME conflict of interest form. Julien H. Park stated that he received travel grants from Biomarin, Amicus and the Recordati Rare Disease foundation. Julia Neugebauer, Karit Reinson, Marcello Bellusci, Omar Hikmat, Enrico Bertini, Manuel Schiff, Anna Ardissone, Niklas Darin, Alejandra Darling, Daria Diodato, Luisa Diogo, Erle Kristensen, Beata Kieć‐Wilk, Maria Carmo Macário, Diego Martinelli, Martina Messina, Mar O'Callaghan, Juan Darío Ortigoza‐Escobar, Margarida Paiva Coelho, Leticia Pías, Jolanta Sykut‐Cegielska, Arnaud Vanlander declare that they have no conflict of interest.

## ETHICS STATEMENT

All procedures followed were in accordance with the ethical standards of the responsible committee on human experimentation (institutional and national) and with the Helsinki Declaration of 1975, as revised in 2000.

## INFORMED CONSENT

This article does not contain any studies with human or animal subjects performed by any of the authors.

## Supporting information


**Appendix S1:** Questionnaire, questionnaire results: the data that support this research are available upon reasonable request.

**FIGURE S1.** Region‐specific availability of the different vitamins and cofactors. This colour‐enhanced table illustrates the regional accessibility of various vitamins and cofactors reported by professionals across different regions. Each row represents a specific geographic region and shows the proportion of professionals in that region who report the availability of this vitamin or cofactor.

**FIGURE S2.** Multiple correspondence analysis exploring the association between geographical location of specialists and use of different vitamins/cofactors. The association between the geographic location (North America, Asia, Europe and Australia/New Zealand) of specialists and their prescribing practices for various vitamins and cofactors show region‐specific prescribing practices.

Visual Abstract

## Data Availability

The raw data supporting this research are available from the corresponding author or first authors upon reasonable request.

## APPENDIX A MetabERN PM‐MD Consortium

### A.1

Anna Ardissone, Child Neurology Unit, Department of Pediatric Neuroscience, Fondazione IRCCS Istituto Neurologico Carlo Besta, Milan, Italy; Niklas Darin, Department of Pediatrics, Institute of Clinical Sciences, University of Gothenburg, Queen Silvia Children's Hospital, Sahlgrenska University Hospital, Gothenburg, Sweden; Alejandra Darling, Metabolic Unit, Pediatric Neurology Department, Institut de Recerca, Hospital Sant Joan de Déu Barcelona, Barcelona, Spain; Movement Disorders Unit, Pediatric Neurology Department, Institut de Recerca, Hospital Sant Joan de Déu Barcelona, Barcelona, Spain; European Reference Network for Rare Neurological Diseases (ERN‐RND), Barcelona, Spain; Daria Diodato, Unit of Neuromuscular and Neurodegenerative Disorders, Bambino Gesù Children's Hospital IRCCS, Rome, Italy; Luisa Diogo, Centro Hospitalar Universitário de Coimbra Centro de Referência de Doenças Hereditárias do Metabolismo Coimbra, Portugal; Erle Kristensen, Department of Clinical Medicine (K1), University of Bergen, Norway; Department of Medical Biochemistry, Oslo University Hospital, Oslo, Norway; Beata Kieć‐Wilk, Unit of Rare Metabolic Diseases Jagiellonian University Medical College, St John Paul II Specialist Hospital, Krakow, Poland; Maria Carmo Macário, Department of Neurology, Coimbra Metabolic Reference Center, Centro Hospitalar e Universitário de Coimbra, Coimbra, Portugal; Diego Martinelli, Unit of Metabolic Diseases and Hepatology, Bambino Gesù Children's Research Hospital, Piazza Sant'Onofrio, 4 ‐ 00165 Rome, Italy; Martina Messina, Mitochondrial Research Group, Genetics and Genomic Medicine Department, UCL Great Ormond Street Institute of Child Health, London, UK; Metabolic Unit, Great Ormond Street Hospital for Children NHS Foundation Trust, London, UK; Mar O'Callaghan, Metabolic Unit, Pediatric Neurology Department, Institut de Recerca, Hospital Sant Joan de Déu Barcelona, Barcelona, Spain; Juan Darío Ortigoza‐Escobar, Movement Disorders Unit, Pediatric Neurology Department, Institut de Recerca, Hospital Sant Joan de Déu Barcelona, Barcelona, Spain; European Reference Network for Rare Neurological Diseases (ERN‐RND), Barcelona, Spain; Biomedical Network Research Centre on Rare Diseases (CIBERER), Instituto de Salud Carlos III, Madrid, Spain; Margarida Paiva Coelho, Reference Center for Metabolic Disorders, Centro Hospitalar do Porto, Largo da Maternidade, S/N, 4450, Porto, Portugal; Leticia Pías, Metabolic Unit, Pediatric Neurology Department, Institut de Recerca, Hospital Sant Joan de Déu Barcelona, Barcelona, Spain; Department of Genetic and Molecular Medicine/IPER, Institut de Recerca, Hospital Sant Joan de Déu Barcelona, Barcelona, Spain; Jolanta Sykut‐Cegielska, Department of Inborn Errors of Metabolism and Pediatrics, Institute of Mother and Child, Warsaw, Poland; Arnaud Vanlander, Department of Paediatrics, Paediatric Neurology and Metabolism, Ghent University Hospital, Ghent, Belgium .
