# Supplementary material for: Current global vitamin and cofactor prescribing practices for primary mitochondrial diseases: Results of a European reference network survey
Source: J Inherit Metab Dis. 2024 Nov 11;48(1):e12805. doi: 10.1002/jimd.12805 (PMC11670042; doi:10.1002/jimd.12805)
Supplement: Supplementary file 1 — Appendix S1: Questionnaire, questionnaire results: the data that support this research are available upon reasonable request. [file JIMD-48-0-s001.pdf]

Section A: Personal details

A1. **Name**

A2. **E-mail address**

A3. **Working centre**

A4. **Working country**

A5. **Type of specialist**

- Pediatric neurologist ☐
- Pediatrician - Inborn Errors of Metabolism specialist ☐
- Adult Neurologist ☐
- Internal medicine doctor ☐
- Ophthalmologist ☐
- Geneticist ☐
- Other ☐

Other

A6. **ERN where you work**

- MetabERN Unit ☐
- EpiCare Unit ☐
- Eye-ERN Unit ☐
- NMD-ERN Unit ☐
- RND-ERN Unit ☐
- Other national reference center but not ERN center ☐
- Other ☐

Other

____

Section B: Patients

B1. **How many patients with genetically defined Primary Mitochondrial Disease (PMD) are you actively following-up?**

- <10 ☐
- 11-50 ☐
- 51-100 ☐
- >100 ☐

B2. **What is the age group of your patients? (mark the ones you follow)**

- Neonates or newborns (birth to 1 month) ☐
- Infants (1 month to 1 year) ☐
- Children (1 year through 17 years) ☐
- Adults (18 years or older) ☐

Section C: Clinical questionnaire

C1. **Do you use vitamins/cofactors to treat primary mitochondrial disorders?**

- Yes ☐
- No ☐

LimeSurvey

C2. Which vitamins/cofactors do you use?

- ☐ Alpha-lipoic acid
- ☐ Arginine
- ☐ Bicarbonate
- ☐ Biotine
- ☐ Carnitine
- ☐ Citrate
- ☐ Citrulline
- ☐ Creatine
- ☐ Cysteine
- ☐ Folinic acid
- ☐ Glutamine
- ☐ Idebenone
- ☐ N-acetylcysteine
- ☐ Nicotinamide riboside
- ☐ Pyruvate
- ☐ Riboflavine
- ☐ Succinate
- ☐ Taurine
- ☐ Thiamine
- ☐ Vitamin C
- ☐ Vitamin E
- ☐ Ubiquinol
- ☐ Ubiquinone
- ☐ Others (please specify which vitamins/cofactors and what for):

Others (please specify which vitamins/cofactors and what for):

LimeSurvey

C3. Alpha-lipoic acid - Mark more precisely

- [ ] In all PMD patients
- [ ] Depending on genotype/phenotype

C4. Genotype/phenotype list (mark the ones in which you use alpha-lipoic acid)

- [ ] Congenital lactic acidosis
- [ ] Suspected/confirmed mitochondrial cardiomyopathy
- [ ] Primary mitochondrial myopathy
- [ ] Benign reversible mitochondrial myopathy
- [ ] Acute liver failure especially in neonates
- [ ] Acute initial presentation of Leigh syndrome spectrum
- [ ] Acute decompensation in Leigh syndrome
- [ ] Chronic Leigh syndrome spectrum
- [ ] MEGDEL
- [ ] Acute stroke-like episode in suspected/confirmed MELAS syndrome
- [ ] Chronic MELAS syndrome
- [ ] MIDD
- [ ] MERRF
- [ ] Pearson syndrome
- [ ] Kearns-Sayre syndrome
- [ ] Mitochondrial optic neuropathy – LHON/ADOA/other cause
- [ ] Mitochondrial depletion syndrome
- [ ] NARP syndrome
- [ ] MNGIE
- [ ] CPEO
- [ ] Confirmed disorder of coenzyme Q10 biosynthesis
- [ ] Confirmed diagnosis of ACAD9 deficiency
- [ ] PDH deficiency
- [ ] PDH E3 deficiency
- [ ] TRMU deficiency

TMEM70 deficiency ☐

Complex I deficiency ☐

Complex II deficiency ☐

Complex III deficiency ☐

Complex IV deficiency ☐

Complex V deficiency ☐

Multiple mitochondrial complex deficiency ☐

Cerebral arginine deficiency ☐

Low CSF 5MTHF ☐

Primary carnitine deficiency ☐

Riboflavin transporter defects ☐

Alpers syndrome ☐

POLG phenotype ☐

DARS2 defect ☐

ETF and ETFDH defects ☐

☐

C5. Arginine - Mark more precisely

In all PMD patients ☐

Depending on genotype/phenotype ☐

C6. Genotype/phenotype list (mark the ones in which you use arginine)

Congenital lactic acidosis ☐

Suspected/confirmed mitochondrial cardiomyopathy ☐

Primary mitochondrial myopathy ☐

Benign reversible mitochondrial myopathy ☐

Acute liver failure especially in neonates ☐

Acute initial presentation of Leigh syndrome spectrum ☐

Acute decompensation in Leigh syndrome ☐

LimeSurvey

Chronic Leigh syndrome spectrum ☐

MEGDEL ☐

Acute stroke-like episode in suspected/confirmed MELAS syndrome ☐

Chronic MELAS syndrome ☐

MIDD ☐

MERRF ☐

Pearson syndrome ☐

Kearns-Sayre syndrome ☐

Mitochondrial optic neuropathy – LHON/ADOA/other cause ☐

Mitochondrial depletion syndrome ☐

NARP syndrome ☐

MNGIE ☐

CPEO ☐

Confirmed disorder of coenzyme Q10 biosynthesis ☐

Confirmed diagnosis of ACAD9 deficiency ☐

PDH deficiency ☐

PDH E3 deficiency ☐

TRMU deficiency ☐

TMEM70 deficiency ☐

Complex I deficiency ☐

Complex II deficiency ☐

Complex III deficiency ☐

Complex IV deficiency ☐

Complex V deficiency ☐

Multiple mitochondrial complex deficiency ☐

Cerebral arginine deficiency ☐

Low CSF 5MTHF ☐

Primary carnitine deficiency ☐

Riboflavin transporter defects ☐

LimeSurvey

Alpers syndrome ☐

POLG phenotype ☐

DARS2 defect ☐

ETF and ETFDH defects ☐

☐

C7. Bicarbonate - Mark more precisely

In all PMD patients ☐

Depending on genotype/phenotype ☐

C8. Genotype/phenotype list (mark the ones in which you use bicarbonate)

Congenital lactic acidosis ☐

Suspected/confirmed mitochondrial cardiomyopathy ☐

Primary mitochondrial myopathy ☐

Benign reversible mitochondrial myopathy ☐

Acute liver failure especially in neonates ☐

Acute initial presentation of Leigh syndrome spectrum ☐

Acute decompensation in Leigh syndrome ☐

Chronic Leigh syndrome spectrum ☐

MEGDEL ☐

Acute stroke-like episode in suspected/confirmed MELAS syndrome ☐

Chronic MELAS syndrome ☐

MIDD ☐

MERRF ☐

Pearson syndrome ☐

Kearns-Sayre syndrome ☐

Mitochondrial optic neuropathy – LHON/ADOA/other cause ☐

Mitochondrial depletion syndrome ☐

NARP syndrome ☐

LimeSurvey

MNGIE ☐

CPEO ☐

Confirmed disorder of coenzyme Q10 biosynthesis ☐

Confirmed diagnosis of ACAD9 deficiency ☐

PDH deficiency ☐

PDH E3 deficiency ☐

TRMU deficiency ☐

TMEM70 deficiency ☐

Complex I deficiency ☐

Complex II deficiency ☐

Complex III deficiency ☐

Complex IV deficiency ☐

Complex V deficiency ☐

Multiple mitochondrial complex deficiency ☐

Cerebral arginine deficiency ☐

Low CSF 5MTHF ☐

Primary carnitine deficiency ☐

Riboflavin transporter defects ☐

Alpers syndrome ☐

POLG phenotype ☐

DARS2 defect ☐

ETF and ETFDH defects ☐

☐

C9. Biotine - Mark more precisely

In all PMD patients ☐

Depending on genotype/phenotype ☐

C10. Genotype/phenotype list (mark the ones in which you use biotine)

- [ ] Congenital lactic acidosis
- [ ] Suspected/confirmed mitochondrial cardiomyopathy
- [ ] Primary mitochondrial myopathy
- [ ] Benign reversible mitochondrial myopathy
- [ ] Acute liver failure especially in neonates
- [ ] Acute initial presentation of Leigh syndrome spectrum
- [ ] Acute decompensation in Leigh syndrome
- [ ] Chronic Leigh syndrome spectrum
- [ ] MEGDEL
- [ ] Acute stroke-like episode in suspected/confirmed MELAS syndrome
- [ ] Chronic MELAS syndrome
- [ ] MIDD
- [ ] MERRF
- [ ] Pearson syndrome
- [ ] Kearns-Sayre syndrome
- [ ] Mitochondrial optic neuropathy – LHON/ADOA/other cause
- [ ] Mitochondrial depletion syndrome
- [ ] NARP syndrome
- [ ] MNGIE
- [ ] CPEO
- [ ] Confirmed disorder of coenzyme Q10 biosynthesis
- [ ] Confirmed diagnosis of ACAD9 deficiency
- [ ] PDH deficiency
- [ ] PDH E3 deficiency
- [ ] TRMU deficiency
- [ ] TMEM70 deficiency
- [ ] Complex I deficiency
- [ ] Complex II deficiency
- [ ] Complex III deficiency

Complex IV deficiency ☐

Complex V deficiency ☐

Multiple mitochondrial complex deficiency ☐

Cerebral arginine deficiency ☐

Low CSF 5MTHF ☐

Primary carnitine deficiency ☐

Riboflavin transporter defects ☐

Alpers syndrome ☐

POLG phenotype ☐

DARS2 defect ☐

ETF and ETFDH defects ☐

☐

C11. Carnitine - Mark more precisely

In all PMD patients ☐

Depending on genotype/phenotype ☐

C12. Genotype/phenotype list (mark the ones in which you use carnitine)

Congenital lactic acidosis ☐

Suspected/confirmed mitochondrial cardiomyopathy ☐

Primary mitochondrial myopathy ☐

Benign reversible mitochondrial myopathy ☐

Acute liver failure especially in neonates ☐

Acute initial presentation of Leigh syndrome spectrum ☐

Acute decompensation in Leigh syndrome ☐

Chronic Leigh syndrome spectrum ☐

MEGDEL ☐

Acute stroke-like episode in suspected/confirmed MELAS syndrome ☐

Chronic MELAS syndrome ☐

LimeSurvey

MIDD ☐

MERRF ☐

Pearson syndrome ☐

Kearns-Sayre syndrome ☐

Mitochondrial optic neuropathy – LHON/ADOA/other cause ☐

Mitochondrial depletion syndrome ☐

NARP syndrome ☐

MNGIE ☐

CPEO ☐

Confirmed disorder of coenzyme Q10 biosynthesis ☐

Confirmed diagnosis of ACAD9 deficiency ☐

PDH deficiency ☐

PDH E3 deficiency ☐

TRMU deficiency ☐

TMEM70 deficiency ☐

Complex I deficiency ☐

Complex II deficiency ☐

Complex III deficiency ☐

Complex IV deficiency ☐

Complex V deficiency ☐

Multiple mitochondrial complex deficiency ☐

Cerebral arginine deficiency ☐

Low CSF 5MTHF ☐

Primary carnitine deficiency ☐

Riboflavin transporter defects ☐

Alpers syndrome ☐

POLG phenotype ☐

DARS2 defect ☐

ETF and ETFDH defects ☐

C13. Citrate - Mark more precisely

- [ ] In all PMD patients
- [ ] Depending on genotype/phenotype

C14. Genotype/phenotype list (mark the ones in which you use citrate)

- [ ] Congenital lactic acidosis
- [ ] Suspected/confirmed mitochondrial cardiomyopathy
- [ ] Primary mitochondrial myopathy
- [ ] Benign reversible mitochondrial myopathy
- [ ] Acute liver failure especially in neonates
- [ ] Acute initial presentation of Leigh syndrome spectrum
- [ ] Acute decompensation in Leigh syndrome
- [ ] Chronic Leigh syndrome spectrum
- [ ] MEGDEL
- [ ] Acute stroke-like episode in suspected/confirmed MELAS syndrome
- [ ] Chronic MELAS syndrome
- [ ] MIDD
- [ ] MERRF
- [ ] Pearson syndrome
- [ ] Kearns-Sayre syndrome
- [ ] Mitochondrial optic neuropathy – LHON/ADOA/other cause
- [ ] Mitochondrial depletion syndrome
- [ ] NARP syndrome
- [ ] MNGIE
- [ ] CPEO
- [ ] Confirmed disorder of coenzyme Q10 biosynthesis
- [ ] Confirmed diagnosis of ACAD9 deficiency

PDH deficiency ☐

PDH E3 deficiency ☐

TRMU deficiency ☐

TMEM70 deficiency ☐

Complex I deficiency ☐

Complex II deficiency ☐

Complex III deficiency ☐

Complex IV deficiency ☐

Complex V deficiency ☐

Multiple mitochondrial complex deficiency ☐

Cerebral arginine deficiency ☐

Low CSF 5MTHF ☐

Primary carnitine deficiency ☐

Riboflavin transporter defects ☐

Alpers syndrome ☐

POLG phenotype ☐

DARS2 defect ☐

ETF and ETFDH defects ☐

☐

C15. Citrulline - Mark more precisely

In all PMD patients ☐

Depending on genotype/phenotype ☐

C16. Genotype/phenotype list (mark the ones in which you use citrulline)

Congenital lactic acidosis ☐

Suspected/confirmed mitochondrial cardiomyopathy ☐

Primary mitochondrial myopathy ☐

Benign reversible mitochondrial myopathy ☐

- [ ] Acute liver failure especially in neonates
- [ ] Acute initial presentation of Leigh syndrome spectrum
- [ ] Acute decompensation in Leigh syndrome
- [ ] Chronic Leigh syndrome spectrum
- [ ] MEGDEL
- [ ] Acute stroke-like episode in suspected/confirmed MELAS syndrome
- [ ] Chronic MELAS syndrome
- [ ] MIDD
- [ ] MERRF
- [ ] Pearson syndrome
- [ ] Kearns-Sayre syndrome
- [ ] Mitochondrial optic neuropathy – LHON/ADOA/other cause
- [ ] Mitochondrial depletion syndrome
- [ ] NARP syndrome
- [ ] MNGIE
- [ ] CPEO
- [ ] Confirmed disorder of coenzyme Q10 biosynthesis
- [ ] Confirmed diagnosis of ACAD9 deficiency
- [ ] PDH deficiency
- [ ] PDH E3 deficiency
- [ ] TRMU deficiency
- [ ] TMEM70 deficiency
- [ ] Complex I deficiency
- [ ] Complex II deficiency
- [ ] Complex III deficiency
- [ ] Complex IV deficiency
- [ ] Complex V deficiency
- [ ] Multiple mitochondrial complex deficiency
- [ ] Cerebral arginine deficiency

Low CSF 5MTHF ☐

Primary carnitine deficiency ☐

Riboflavin transporter defects ☐

Alpers syndrome ☐

POLG phenotype ☐

DARS2 defect ☐

ETF and ETFDH defects ☐

☐

C17. Creatine - Mark more precisely

In all PMD patients ☐

Depending on genotype/phenotype ☐

C18. Genotype/phenotype list (mark the ones in which you use creatine)

Congenital lactic acidosis ☐

Suspected/confirmed mitochondrial cardiomyopathy ☐

Primary mitochondrial myopathy ☐

Benign reversible mitochondrial myopathy ☐

Acute liver failure especially in neonates ☐

Acute initial presentation of Leigh syndrome spectrum ☐

Acute decompensation in Leigh syndrome ☐

Chronic Leigh syndrome spectrum ☐

MEGDEL ☐

Acute stroke-like episode in suspected/confirmed MELAS syndrome ☐

Chronic MELAS syndrome ☐

MIDD ☐

MERRF ☐

Pearson syndrome ☐

Kearns-Sayre syndrome ☐

LimeSurvey

Mitochondrial optic neuropathy – LHON/ADOA/other cause ☐

Mitochondrial depletion syndrome ☐

NARP syndrome ☐

MNGIE ☐

CPEO ☐

Confirmed disorder of coenzyme Q10 biosynthesis ☐

Confirmed diagnosis of ACAD9 deficiency ☐

PDH deficiency ☐

PDH E3 deficiency ☐

TRMU deficiency ☐

TMEM70 deficiency ☐

Complex I deficiency ☐

Complex II deficiency ☐

Complex III deficiency ☐

Complex IV deficiency ☐

Complex V deficiency ☐

Multiple mitochondrial complex deficiency ☐

Cerebral arginine deficiency ☐

Low CSF 5MTHF ☐

Primary carnitine deficiency ☐

Riboflavin transporter defects ☐

Alpers syndrome ☐

POLG phenotype ☐

DARS2 defect ☐

ETF and ETFDH defects ☐

☐

LimeSurvey

C19. **Cysteine - Mark more precisely**

- [ ] In all PMD patients
- [ ] Depending on genotype/phenotype

C20. **Genotype/phenotype list (mark the ones in which you use cysteine)**

- [ ] Congenital lactic acidosis
- [ ] Suspected/confirmed mitochondrial cardiomyopathy
- [ ] Primary mitochondrial myopathy
- [ ] Benign reversible mitochondrial myopathy
- [ ] Acute liver failure especially in neonates
- [ ] Acute initial presentation of Leigh syndrome spectrum
- [ ] Acute decompensation in Leigh syndrome
- [ ] Chronic Leigh syndrome spectrum
- [ ] MEGDEL
- [ ] Acute stroke-like episode in suspected/confirmed MELAS syndrome
- [ ] Chronic MELAS syndrome
- [ ] MIDD
- [ ] MERRF
- [ ] Pearson syndrome
- [ ] Kearns-Sayre syndrome
- [ ] Mitochondrial optic neuropathy – LHON/ADOA/other cause
- [ ] Mitochondrial depletion syndrome
- [ ] NARP syndrome
- [ ] MNGIE
- [ ] CPEO
- [ ] Confirmed disorder of coenzyme Q10 biosynthesis
- [ ] Confirmed diagnosis of ACAD9 deficiency
- [ ] PDH deficiency
- [ ] PDH E3 deficiency
- [ ] TRMU deficiency
- [ ] TMEM70 deficiency

LimeSurvey

Complex I deficiency ☐

Complex II deficiency ☐

Complex III deficiency ☐

Complex IV deficiency ☐

Complex V deficiency ☐

Multiple mitochondrial complex deficiency ☐

Cerebral arginine deficiency ☐

Low CSF 5MTHF ☐

Primary carnitine deficiency ☐

Riboflavin transporter defects ☐

Alpers syndrome ☐

POLG phenotype ☐

DARS2 defect ☐

ETF and ETFDH defects ☐

☐

C21. Folinic acid - Mark more precisely

In all PMD patients ☐

Depending on genotype/phenotype ☐

C22. Genotype/phenotype list (mark the ones in which you use folinic acid)

Congenital lactic acidosis ☐

Suspected/confirmed mitochondrial cardiomyopathy ☐

Primary mitochondrial myopathy ☐

Benign reversible mitochondrial myopathy ☐

Acute liver failure especially in neonates ☐

Acute initial presentation of Leigh syndrome spectrum ☐

Acute decompensation in Leigh syndrome ☐

Chronic Leigh syndrome spectrum ☐

LimeSurvey

MEGDEL ☐

Acute stroke-like episode in suspected/confirmed MELAS syndrome ☐

Chronic MELAS syndrome ☐

MIDD ☐

MERRF ☐

Pearson syndrome ☐

Kearns-Sayre syndrome ☐

Mitochondrial optic neuropathy – LHON/ADOA/other cause ☐

Mitochondrial depletion syndrome ☐

NARP syndrome ☐

MNGIE ☐

CPEO ☐

Confirmed disorder of coenzyme Q10 biosynthesis ☐

Confirmed diagnosis of ACAD9 deficiency ☐

PDH deficiency ☐

PDH E3 deficiency ☐

TRMU deficiency ☐

TMEM70 deficiency ☐

Complex I deficiency ☐

Complex II deficiency ☐

Complex III deficiency ☐

Complex IV deficiency ☐

Complex V deficiency ☐

Multiple mitochondrial complex deficiency ☐

Cerebral arginine deficiency ☐

Low CSF 5MTHF ☐

Primary carnitine deficiency ☐

Riboflavin transporter defects ☐

Alpers syndrome ☐

POLG phenotype ☐

DARS2 defect ☐

ETF and ETFDH defects ☐

☐

C23. Glutamine - Mark more precisely

In all PMD patients ☐

Depending on genotype/phenotype ☐

C24. Genotype/phenotype list (mark the ones in which you use glutamine)

Congenital lactic acidosis ☐

Suspected/confirmed mitochondrial cardiomyopathy ☐

Primary mitochondrial myopathy ☐

Benign reversible mitochondrial myopathy ☐

Acute liver failure especially in neonates ☐

Acute initial presentation of Leigh syndrome spectrum ☐

Acute decompensation in Leigh syndrome ☐

Chronic Leigh syndrome spectrum ☐

MEGDEL ☐

Acute stroke-like episode in suspected/confirmed MELAS syndrome ☐

Chronic MELAS syndrome ☐

MIDD ☐

MERRF ☐

Pearson syndrome ☐

Kearns-Sayre syndrome ☐

Mitochondrial optic neuropathy – LHON/ADOA/other cause ☐

Mitochondrial depletion syndrome ☐

NARP syndrome ☐

MNGIE ☐

CPEO ☐

Confirmed disorder of coenzyme Q10 biosynthesis ☐

Confirmed diagnosis of ACAD9 deficiency ☐

PDH deficiency ☐

PDH E3 deficiency ☐

TRMU deficiency ☐

TMEM70 deficiency ☐

Complex I deficiency ☐

Complex II deficiency ☐

Complex III deficiency ☐

Complex IV deficiency ☐

Complex V deficiency ☐

Multiple mitochondrial complex deficiency ☐

Cerebral arginine deficiency ☐

Low CSF 5MTHF ☐

Primary carnitine deficiency ☐

Riboflavin transporter defects ☐

Alpers syndrome ☐

POLG phenotype ☐

DARS2 defect ☐

ETF and ETFDH defects ☐

☐

C25. Idebenone - Mark more precisely

In all PMD patients ☐

Depending on genotype/phenotype ☐

C26. Genotype/phenotype list (mark the ones in which you use idebenone)

Congenital lactic acidosis ☐

LimeSurvey

- [ ] Suspected/confirmed mitochondrial cardiomyopathy
- [ ] Primary mitochondrial myopathy
- [ ] Benign reversible mitochondrial myopathy
- [ ] Acute liver failure especially in neonates
- [ ] Acute initial presentation of Leigh syndrome spectrum
- [ ] Acute decompensation in Leigh syndrome
- [ ] Chronic Leigh syndrome spectrum
- [ ] MEGDEL
- [ ] Acute stroke-like episode in suspected/confirmed MELAS syndrome
- [ ] Chronic MELAS syndrome
- [ ] MIDD
- [ ] MERRF
- [ ] Pearson syndrome
- [ ] Kearns-Sayre syndrome
- [ ] Mitochondrial optic neuropathy – LHON/ADOA/other cause
- [ ] Mitochondrial depletion syndrome
- [ ] NARP syndrome
- [ ] MNGIE
- [ ] CPEO
- [ ] Confirmed disorder of coenzyme Q10 biosynthesis
- [ ] Confirmed diagnosis of ACAD9 deficiency
- [ ] PDH deficiency
- [ ] PDH E3 deficiency
- [ ] TRMU deficiency
- [ ] TMEM70 deficiency
- [ ] Complex I deficiency
- [ ] Complex II deficiency
- [ ] Complex III deficiency
- [ ] Complex IV deficiency

LimeSurvey

Complex V deficiency ☐

Multiple mitochondrial complex deficiency ☐

Cerebral arginine deficiency ☐

Low CSF 5MTHF ☐

Primary carnitine deficiency ☐

Riboflavin transporter defects ☐

Alpers syndrome ☐

POLG phenotype ☐

DARS2 defect ☐

ETF and ETFDH defects ☐

☐

C27. N-acetylcysteine - Mark more precisely

In all PMD patients ☐

Depending on genotype/phenotype ☐

C28. Genotype/phenotype list (mark the ones in which you use N-acetylcysteine)

Congenital lactic acidosis ☐

Suspected/confirmed mitochondrial cardiomyopathy ☐

Primary mitochondrial myopathy ☐

Benign reversible mitochondrial myopathy ☐

Acute liver failure especially in neonates ☐

Acute initial presentation of Leigh syndrome spectrum ☐

Acute decompensation in Leigh syndrome ☐

Chronic Leigh syndrome spectrum ☐

MEGDEL ☐

Acute stroke-like episode in suspected/confirmed MELAS syndrome ☐

Chronic MELAS syndrome ☐

LimeSurvey

- [ ] MIDD
- [ ] MERRF
- [ ] Pearson syndrome
- [ ] Kearns-Sayre syndrome
- [ ] Mitochondrial optic neuropathy – LHON/ADOA/other cause
- [ ] Mitochondrial depletion syndrome
- [ ] NARP syndrome
- [ ] MNGIE
- [ ] CPEO
- [ ] Confirmed disorder of coenzyme Q10 biosynthesis
- [ ] Confirmed diagnosis of ACAD9 deficiency
- [ ] PDH deficiency
- [ ] PDH E3 deficiency
- [ ] TRMU deficiency
- [ ] TMEM70 deficiency
- [ ] Complex I deficiency
- [ ] Complex II deficiency
- [ ] Complex III deficiency
- [ ] Complex IV deficiency
- [ ] Complex V deficiency
- [ ] Multiple mitochondrial complex deficiency
- [ ] Cerebral arginine deficiency
- [ ] Low CSF 5MTHF
- [ ] Primary carnitine deficiency
- [ ] Riboflavin transporter defects
- [ ] Alpers syndrome
- [ ] POLG phenotype
- [ ] DARS2 defect
- [ ] ETF and ETFDH defects

C29. **Nicotinamide-riboside - Mark more precisely**

- [ ] In all PMD patients
- [ ] Depending on genotype/phenotype

C30. **Genotype/phenotype list (mark the ones in which you use nicotinamide-riboside)**

- [ ] Congenital lactic acidosis
- [ ] Suspected/confirmed mitochondrial cardiomyopathy
- [ ] Primary mitochondrial myopathy
- [ ] Benign reversible mitochondrial myopathy
- [ ] Acute liver failure especially in neonates
- [ ] Acute initial presentation of Leigh syndrome spectrum
- [ ] Acute decompensation in Leigh syndrome
- [ ] Chronic Leigh syndrome spectrum
- [ ] MEGDEL
- [ ] Acute stroke-like episode in suspected/confirmed MELAS syndrome
- [ ] Chronic MELAS syndrome
- [ ] MIDD
- [ ] MERRF
- [ ] Pearson syndrome
- [ ] Kearns-Sayre syndrome
- [ ] Mitochondrial optic neuropathy – LHON/ADOA/other cause
- [ ] Mitochondrial depletion syndrome
- [ ] NARP syndrome
- [ ] MNGIE
- [ ] CPEO
- [ ] Confirmed disorder of coenzyme Q10 biosynthesis

Confirmed diagnosis of ACAD9 deficiency ☐

PDH deficiency ☐

PDH E3 deficiency ☐

TRMU deficiency ☐

TMEM70 deficiency ☐

Complex I deficiency ☐

Complex II deficiency ☐

Complex III deficiency ☐

Complex IV deficiency ☐

Complex V deficiency ☐

Multiple mitochondrial complex deficiency ☐

Cerebral arginine deficiency ☐

Low CSF 5MTHF ☐

Primary carnitine deficiency ☐

Riboflavin transporter defects ☐

Alpers syndrome ☐

POLG phenotype ☐

DARS2 defect ☐

ETF and ETFDH defects ☐

☐

C31. Pyruvate - Mark more precisely

In all PMD patients ☐

Depending on genotype/phenotype ☐

C32. Genotype/phenotype list (mark the ones in which you use pyruvate)

Congenital lactic acidosis ☐

Suspected/confirmed mitochondrial cardiomyopathy ☐

Primary mitochondrial myopathy ☐

LimeSurvey

- [ ] Benign reversible mitochondrial myopathy
- [ ] Acute liver failure especially in neonates
- [ ] Acute initial presentation of Leigh syndrome spectrum
- [ ] Acute decompensation in Leigh syndrome
- [ ] Chronic Leigh syndrome spectrum
- [ ] MEGDEL
- [ ] Acute stroke-like episode in suspected/confirmed MELAS syndrome
- [ ] Chronic MELAS syndrome
- [ ] MIDD
- [ ] MERRF
- [ ] Pearson syndrome
- [ ] Kearns-Sayre syndrome
- [ ] Mitochondrial optic neuropathy – LHON/ADOA/other cause
- [ ] Mitochondrial depletion syndrome
- [ ] NARP syndrome
- [ ] MNGIE
- [ ] CPEO
- [ ] Confirmed disorder of coenzyme Q10 biosynthesis
- [ ] Confirmed diagnosis of ACAD9 deficiency
- [ ] PDH deficiency
- [ ] PDH E3 deficiency
- [ ] TRMU deficiency
- [ ] TMEM70 deficiency
- [ ] Complex I deficiency
- [ ] Complex II deficiency
- [ ] Complex III deficiency
- [ ] Complex IV deficiency
- [ ] Complex V deficiency
- [ ] Multiple mitochondrial complex deficiency

LimeSurvey

Cerebral arginine deficiency ☐

Low CSF 5MTHF ☐

Primary carnitine deficiency ☐

Riboflavin transporter defects ☐

Alpers syndrome ☐

POLG phenotype ☐

DARS2 defect ☐

ETF and ETFDH defects ☐

☐

C33. Riboflavine - Mark more precisely

In all PMD patients ☐

Depending on genotype/phenotype ☐

C34. Genotype/phenotype list (mark the ones in which you use riboflavine)

Congenital lactic acidosis ☐

Suspected/confirmed mitochondrial cardiomyopathy ☐

Primary mitochondrial myopathy ☐

Benign reversible mitochondrial myopathy ☐

Acute liver failure especially in neonates ☐

Acute initial presentation of Leigh syndrome spectrum ☐

Acute decompensation in Leigh syndrome ☐

Chronic Leigh syndrome spectrum ☐

MEGDEL ☐

Acute stroke-like episode in suspected/confirmed MELAS syndrome ☐

Chronic MELAS syndrome ☐

MIDD ☐

MERRF ☐

Pearson syndrome ☐

LimeSurvey

- [ ] Kearns-Sayre syndrome
- [ ] Mitochondrial optic neuropathy – LHON/ADOA/other cause
- [ ] Mitochondrial depletion syndrome
- [ ] NARP syndrome
- [ ] MNGIE
- [ ] CPEO
- [ ] Confirmed disorder of coenzyme Q10 biosynthesis
- [ ] Confirmed diagnosis of ACAD9 deficiency
- [ ] PDH deficiency
- [ ] PDH E3 deficiency
- [ ] TRMU deficiency
- [ ] TMEM70 deficiency
- [ ] Complex I deficiency
- [ ] Complex II deficiency
- [ ] Complex III deficiency
- [ ] Complex IV deficiency
- [ ] Complex V deficiency
- [ ] Multiple mitochondrial complex deficiency
- [ ] Cerebral arginine deficiency
- [ ] Low CSF 5MTHF
- [ ] Primary carnitine deficiency
- [ ] Riboflavin transporter defects
- [ ] Alpers syndrome
- [ ] POLG phenotype
- [ ] DARS2 defect
- [ ] ETF and ETFDH defects

LimeSurvey

C35. **Succinate - Mark more precisely**

- In all PMD patients ☐
- Depending on genotype/phenotype ☐

C36. **Genotype/phenotype list (mark the ones in which you use succinate)**

- Congenital lactic acidosis ☐
- Suspected/confirmed mitochondrial cardiomyopathy ☐
- Primary mitochondrial myopathy ☐
- Benign reversible mitochondrial myopathy ☐
- Acute liver failure especially in neonates ☐
- Acute initial presentation of Leigh syndrome spectrum ☐
- Acute decompensation in Leigh syndrome ☐
- Chronic Leigh syndrome spectrum ☐
- MEGDEL ☐
- Acute stroke-like episode in suspected/confirmed MELAS syndrome ☐
- Chronic MELAS syndrome ☐
- MIDD ☐
- MERRF ☐
- Pearson syndrome ☐
- Kearns-Sayre syndrome ☐
- Mitochondrial optic neuropathy – LHON/ADOA/other cause ☐
- Mitochondrial depletion syndrome ☐
- NARP syndrome ☐
- MNGIE ☐
- CPEO ☐
- Confirmed disorder of coenzyme Q10 biosynthesis ☐
- Confirmed diagnosis of ACAD9 deficiency ☐

LimeSurvey

PDH deficiency ☐

PDH E3 deficiency ☐

TRMU deficiency ☐

TMEM70 deficiency ☐

Complex I deficiency ☐

Complex II deficiency ☐

Complex III deficiency ☐

Complex IV deficiency ☐

Complex V deficiency ☐

Multiple mitochondrial complex deficiency ☐

Cerebral arginine deficiency ☐

Low CSF 5MTHF ☐

Primary carnitine deficiency ☐

Riboflavin transporter defects ☐

Alpers syndrome ☐

POLG phenotype ☐

DARS2 defect ☐

ETF and ETFDH defects ☐

☐

C37. Taurine - Mark more precisely

In all PMD patients ☐

Depending on genotype/phenotype ☐

C38. Genotype/phenotype list (mark the ones in which you use taurine)

Congenital lactic acidosis ☐

Suspected/confirmed mitochondrial cardiomyopathy ☐

Primary mitochondrial myopathy ☐

Benign reversible mitochondrial myopathy ☐

LimeSurvey

- [ ] Acute liver failure especially in neonates
- [ ] Acute initial presentation of Leigh syndrome spectrum
- [ ] Acute decompensation in Leigh syndrome
- [ ] Chronic Leigh syndrome spectrum
- [ ] MEGDEL
- [ ] Acute stroke-like episode in suspected/confirmed MELAS syndrome
- [ ] Chronic MELAS syndrome
- [ ] MIDD
- [ ] MERRF
- [ ] Pearson syndrome
- [ ] Kearns-Sayre syndrome
- [ ] Mitochondrial optic neuropathy – LHON/ADOA/other cause
- [ ] Mitochondrial depletion syndrome
- [ ] NARP syndrome
- [ ] MNGIE
- [ ] CPEO
- [ ] Confirmed disorder of coenzyme Q10 biosynthesis
- [ ] Confirmed diagnosis of ACAD9 deficiency
- [ ] PDH deficiency
- [ ] PDH E3 deficiency
- [ ] TRMU deficiency
- [ ] TMEM70 deficiency
- [ ] Complex I deficiency
- [ ] Complex II deficiency
- [ ] Complex III deficiency
- [ ] Complex IV deficiency
- [ ] Complex V deficiency
- [ ] Multiple mitochondrial complex deficiency
- [ ] Cerebral arginine deficiency

Low CSF 5MTHF ☐

Primary carnitine deficiency ☐

Riboflavin transporter defects ☐

Alpers syndrome ☐

POLG phenotype ☐

DARS2 defect ☐

ETF and ETFDH defects ☐

☐

C39. Thiamine - Mark more precisely

In all PMD patients ☐

Depending on genotype/phenotype ☐

C40. Genotype/phenotype list (mark the ones in which you use thiamine)

Congenital lactic acidosis ☐

Suspected/confirmed mitochondrial cardiomyopathy ☐

Primary mitochondrial myopathy ☐

Benign reversible mitochondrial myopathy ☐

Acute liver failure especially in neonates ☐

Acute initial presentation of Leigh syndrome spectrum ☐

Acute decompensation in Leigh syndrome ☐

Chronic Leigh syndrome spectrum ☐

MEGDEL ☐

Acute stroke-like episode in suspected/confirmed MELAS syndrome ☐

Chronic MELAS syndrome ☐

MIDD ☐

MERRF ☐

Pearson syndrome ☐

Kearns-Sayre syndrome ☐

LimeSurvey

Mitochondrial optic neuropathy – LHON/ADOA/other cause ☐

Mitochondrial depletion syndrome ☐

NARP syndrome ☐

MNGIE ☐

CPEO ☐

Confirmed disorder of coenzyme Q10 biosynthesis ☐

Confirmed diagnosis of ACAD9 deficiency ☐

PDH deficiency ☐

PDH E3 deficiency ☐

TRMU deficiency ☐

TMEM70 deficiency ☐

Complex I deficiency ☐

Complex II deficiency ☐

Complex III deficiency ☐

Complex IV deficiency ☐

Complex V deficiency ☐

Multiple mitochondrial complex deficiency ☐

Cerebral arginine deficiency ☐

Low CSF 5MTHF ☐

Primary carnitine deficiency ☐

Riboflavin transporter defects ☐

Alpers syndrome ☐

POLG phenotype ☐

DARS2 defect ☐

ETF and ETFDH defects ☐

☐

C41. Vitamin C - Mark more precisely

In all PMD patients ☐

Depending on genotype/phenotype ☐

C42. Genotype/phenotype list (mark the ones in which you use vitamin C)

Congenital lactic acidosis ☐

Suspected/confirmed mitochondrial cardiomyopathy ☐

Primary mitochondrial myopathy ☐

Benign reversible mitochondrial myopathy ☐

Acute liver failure especially in neonates ☐

Acute initial presentation of Leigh syndrome spectrum ☐

Acute decompensation in Leigh syndrome ☐

Chronic Leigh syndrome spectrum ☐

MEGDEL ☐

Acute stroke-like episode in suspected/confirmed MELAS syndrome ☐

Chronic MELAS syndrome ☐

MIDD ☐

MERRF ☐

Pearson syndrome ☐

Kearns-Sayre syndrome ☐

Mitochondrial optic neuropathy – LHON/ADOA/other cause ☐

Mitochondrial depletion syndrome ☐

NARP syndrome ☐

MNGIE ☐

CPEO ☐

Confirmed disorder of coenzyme Q10 biosynthesis ☐

Confirmed diagnosis of ACAD9 deficiency ☐

PDH deficiency ☐

PDH E3 deficiency ☐

TRMU deficiency ☐

TMEM70 deficiency ☐

LimeSurvey

Complex I deficiency ☐

Complex II deficiency ☐

Complex III deficiency ☐

Complex IV deficiency ☐

Complex V deficiency ☐

Multiple mitochondrial complex deficiency ☐

Cerebral arginine deficiency ☐

Low CSF 5MTHF ☐

Primary carnitine deficiency ☐

Riboflavin transporter defects ☐

Alpers syndrome ☐

POLG phenotype ☐

DARS2 defect ☐

ETF and ETFDH defects ☐

☐

C43. Vitamin E - Mark more precisely

In all PMD patients ☐

Depending on genotype/phenotype ☐

C44. Genotype/phenotype list (mark the ones in which you use vitamin E)

Congenital lactic acidosis ☐

Suspected/confirmed mitochondrial cardiomyopathy ☐

Primary mitochondrial myopathy ☐

Benign reversible mitochondrial myopathy ☐

Acute liver failure especially in neonates ☐

Acute initial presentation of Leigh syndrome spectrum ☐

Acute decompensation in Leigh syndrome ☐

Chronic Leigh syndrome spectrum ☐

LimeSurvey

MEGDEL ☐

Acute stroke-like episode in suspected/confirmed MELAS syndrome ☐

Chronic MELAS syndrome ☐

MIDD ☐

MERRF ☐

Pearson syndrome ☐

Kearns-Sayre syndrome ☐

Mitochondrial optic neuropathy – LHON/ADOA/other cause ☐

Mitochondrial depletion syndrome ☐

NARP syndrome ☐

MNGIE ☐

CPEO ☐

Confirmed disorder of coenzyme Q10 biosynthesis ☐

Confirmed diagnosis of ACAD9 deficiency ☐

PDH deficiency ☐

PDH E3 deficiency ☐

TRMU deficiency ☐

TMEM70 deficiency ☐

Complex I deficiency ☐

Complex II deficiency ☐

Complex III deficiency ☐

Complex IV deficiency ☐

Complex V deficiency ☐

Multiple mitochondrial complex deficiency ☐

Cerebral arginine deficiency ☐

Low CSF 5MTHF ☐

Primary carnitine deficiency ☐

Riboflavin transporter defects ☐

Alpers syndrome ☐

POLG phenotype ☐

DARS2 defect ☐

ETF and ETFDH defects ☐

☐

C45. Ubiquinol - Mark more precisely

In all PMD patients ☐

Depending on genotype/phenotype ☐

C46. Genotype/phenotype list (mark the ones in which you use ubiquinol)

Congenital lactic acidosis ☐

Suspected/confirmed mitochondrial cardiomyopathy ☐

Primary mitochondrial myopathy ☐

Benign reversible mitochondrial myopathy ☐

Acute liver failure especially in neonates ☐

Acute initial presentation of Leigh syndrome spectrum ☐

Acute decompensation in Leigh syndrome ☐

Chronic Leigh syndrome spectrum ☐

MEGDEL ☐

Acute stroke-like episode in suspected/confirmed MELAS syndrome ☐

Chronic MELAS syndrome ☐

MIDD ☐

MERRF ☐

Pearson syndrome ☐

Kearns-Sayre syndrome ☐

Mitochondrial optic neuropathy – LHON/ADOA/other cause ☐

Mitochondrial depletion syndrome ☐

NARP syndrome ☐

MNGIE ☐

LimeSurvey

CPEO ☐

Confirmed disorder of coenzyme Q10 biosynthesis ☐

Confirmed diagnosis of ACAD9 deficiency ☐

PDH deficiency ☐

PDH E3 deficiency ☐

TRMU deficiency ☐

TMEM70 deficiency ☐

Complex I deficiency ☐

Complex II deficiency ☐

Complex III deficiency ☐

Complex IV deficiency ☐

Complex V deficiency ☐

Multiple mitochondrial complex deficiency ☐

Cerebral arginine deficiency ☐

Low CSF 5MTHF ☐

Primary carnitine deficiency ☐

Riboflavin transporter defects ☐

Alpers syndrome ☐

POLG phenotype ☐

DARS2 defect ☐

ETF and ETFDH defects ☐

☐

C47. Ubiquinone - Mark more precisely

In all PMD patients ☐

Depending on genotype/phenotype ☐

C48. Genotype/phenotype list (mark the ones in which you use ubiquinone)

Congenital lactic acidosis ☐

LimeSurvey

- [ ] Suspected/confirmed mitochondrial cardiomyopathy
- [ ] Primary mitochondrial myopathy
- [ ] Benign reversible mitochondrial myopathy
- [ ] Acute liver failure especially in neonates
- [ ] Acute initial presentation of Leigh syndrome spectrum
- [ ] Acute decompensation in Leigh syndrome
- [ ] Chronic Leigh syndrome spectrum
- [ ] MEGDEL
- [ ] Acute stroke-like episode in suspected/confirmed MELAS syndrome
- [ ] Chronic MELAS syndrome
- [ ] MIDD
- [ ] MERRF
- [ ] Pearson syndrome
- [ ] Kearns-Sayre syndrome
- [ ] Mitochondrial optic neuropathy – LHON/ADOA/other cause
- [ ] Mitochondrial depletion syndrome
- [ ] NARP syndrome
- [ ] MNGIE
- [ ] CPEO
- [ ] Confirmed disorder of coenzyme Q10 biosynthesis
- [ ] Confirmed diagnosis of ACAD9 deficiency
- [ ] PDH deficiency
- [ ] PDH E3 deficiency
- [ ] TRMU deficiency
- [ ] TMEM70 deficiency
- [ ] Complex I deficiency
- [ ] Complex II deficiency
- [ ] Complex III deficiency
- [ ] Complex IV deficiency

Complex V deficiency ☐

Multiple mitochondrial complex deficiency ☐

Cerebral arginine deficiency ☐

Low CSF 5MTHF ☐

Primary carnitine deficiency ☐

Riboflavin transporter defects ☐

Alpers syndrome ☐

POLG phenotype ☐

DARS2 defect ☐

ETF and ETFDH defects ☐

☐

Section D: Availability and compensation

D1. Which of these vitamins/cofactors are available in your country?

Alpha-lipoic acid ☐

Arginine ☐

Bicarbonate ☐

Biotine ☐

Carnitine ☐

Citrate ☐

Citrulline ☐

Creatine ☐

Cysteine ☐

Folinic acid ☐

Glutamine ☐

Idebenone ☐

N-acetylcysteine ☐

Nicotinamide riboside ☐

Pyruvate ☐

Riboflavine ☐

Succinate ☐

Taurine ☐

Thiamine ☐

Vitamin C ☐

Vitamin E ☐

Ubiquinol ☐

Ubiquinone ☐

All of the above ☐

Others: ☐

Others:

D2. Are there any other vitamins/cofactors available in your country?

D3. Which of these vitamins/cofactors are financially compensated for the patient?

Alpha-lipoic acid ☐

Arginine ☐

Bicarbonate ☐

Biotine ☐

Carnitine ☐

Citrate ☐

Citrulline ☐

Creatine ☐

Cysteine ☐

Folinic acid ☐

Glutamine ☐

Idebenone ☐

N-acetylcysteine ☐

Nicotinamide riboside ☐

Pyruvate ☐

Riboflavine ☐

Succinate ☐

Taurine ☐

Thiamine ☐

Vitamin C ☐

Vitamin E ☐

Ubiquinol ☐

Ubiquinone ☐

All of the above ☐

Others: ☐

Others:

D4. Are there any other vitamins/cofactors compensated in your country?

Section E: Treatment protocols

E1. Does your centre/hospital/department have general treatment recommendations/protocol or do you recommend treatment based on personal reading/experience?

My centre/hospital/department has general treatment recommendations/protocol ☐

I recommend treatment based on personal reading/experience ☐

E2. Please share your centre/hospital/department's treatment protocol.
