# Supplementary figures and images for: Current global vitamin and cofactor prescribing practices for primary mitochondrial diseases: Results of a European reference network survey
Source: J Inherit Metab Dis. 2024 Nov 11;48(1):e12805. doi: 10.1002/jimd.12805 (PMC11670042; doi:10.1002/jimd.12805)

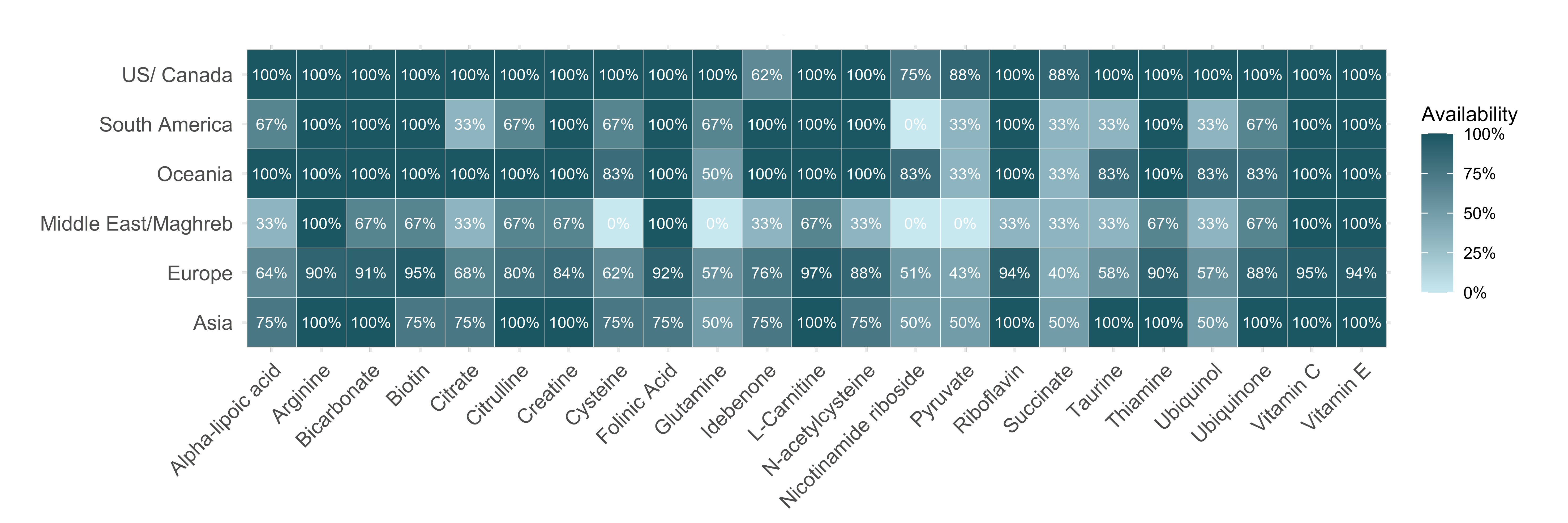

Supplement: Supplementary file 2 — FIGURE S1. Region‐specific availability of the different vitamins and cofactors. This colour‐enhanced table illustrates the regional accessibility of various vitamins and cofactors reported by professionals across different regions. Each row represents a specific geographic region and shows the proportion of professionals in that region who report the availability of this vitamin or cofactor. [file JIMD-48-0-s004.tiff]

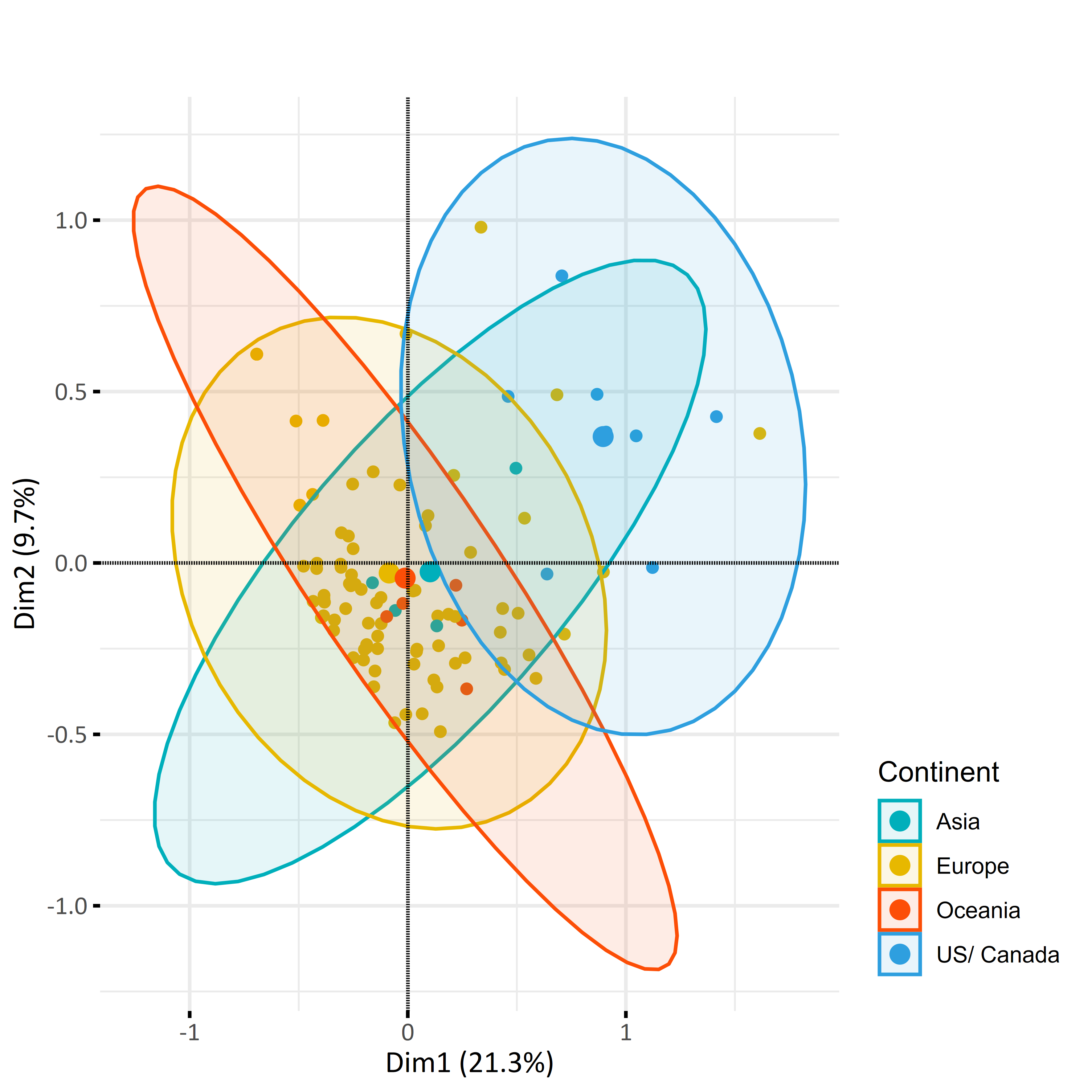

Supplement: Supplementary file 3 — FIGURE S2. Multiple correspondence analysis exploring the association between geographical location of specialists and use of different vitamins/cofactors. The association between the geographic location (North America, Asia, Europe and Australia/New Zealand) of specialists and their prescribing practices for various vitamins and cofactors show region‐specific prescribing practices. [file JIMD-48-0-s002.tiff]

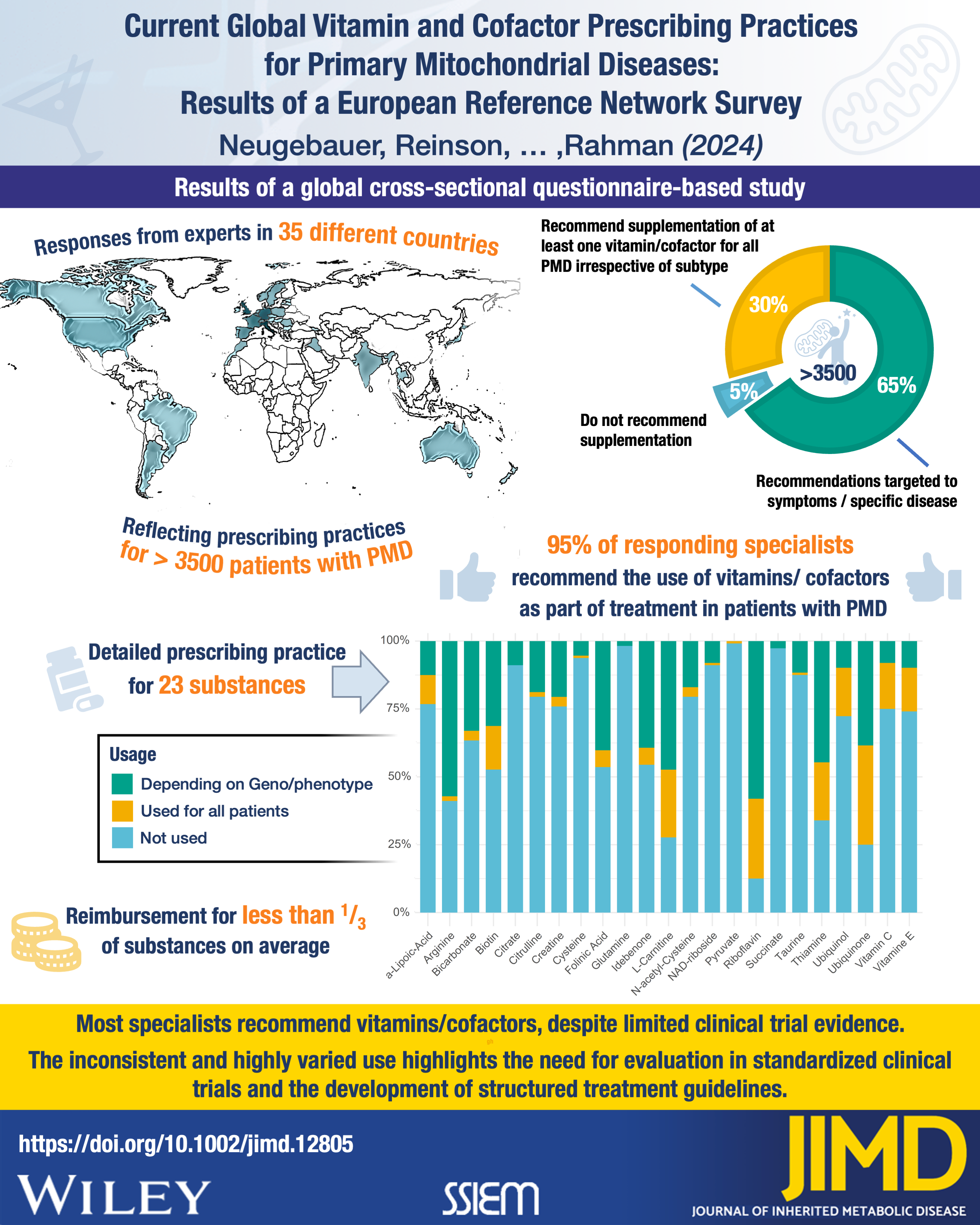

Supplement: Supplementary file 4 — Visual Abstract [file JIMD-48-0-s003.png]
